# Angiogenesis depends upon EPHB4-mediated export of collagen IV from vascular endothelial cells

**DOI:** 10.1172/jci.insight.156928

**Published:** 2022-02-22

**Authors:** Di Chen, Elizabeth D. Hughes, Thomas L. Saunders, Jiangping Wu, Magda N. Hernandez Vasquez, Taija Makinen, Philip D. King

**Affiliations:** 1 Department of Microbiology and Immunology,; 2 Transgenic Animal Model Core, and; 3 Department of Internal Medicine, University of Michigan Medical School, Ann Arbor, Michigan, USA.; 4 Research Centre, Center Hospital of the University of Montréal (CRCHUM), Montréal, Québec, Canada.; 5 Department of Immunology, Genetics and Pathology, Uppsala University, Uppsala, Sweden.

**Keywords:** Angiogenesis, Vascular Biology, Endothelial cells, Signal transduction

## Abstract

Capillary malformation-arteriovenous malformation (CM-AVM) is a blood vascular anomaly caused by inherited loss-of-function mutations in *RASA1* or *EPHB4* genes, which encode p120 Ras GTPase-activating protein (p120 RasGAP/RASA1) and Ephrin receptor B4 (EPHB4). However, whether RASA1 and EPHB4 function in the same molecular signaling pathway to regulate the blood vasculature is uncertain. Here, we show that induced endothelial cell–specific (EC-specific) disruption of *Ephb4* in mice resulted in accumulation of collagen IV in the EC ER, leading to EC apoptotic death and defective developmental, neonatal, and pathological angiogenesis, as reported previously in induced EC-specific RASA1-deficient mice. Moreover, defects in angiogenic responses in EPHB4-deficient mice could be rescued by drugs that inhibit signaling through the Ras pathway and drugs that promote collagen IV export from the ER. However, EPHB4-mutant mice that expressed a form of EPHB4 that is unable to physically engage RASA1 but retains protein tyrosine kinase activity showed normal angiogenic responses. These findings provide strong evidence that RASA1 and EPHB4 function in the same signaling pathway to protect against the development of CM-AVM independent of physical interaction and have important implications for possible means of treatment of this disease.

## Introduction

Capillary malformation-arteriovenous malformation (CM-AVM) is an inherited autosomal dominant blood vascular disorder in humans that affects 1:10,000 to 1:100,000 individuals (1–4). The pathognomonic feature of CM-AVM is the presence of 1 or more cutaneous CMs. However, in approximately one-third of patients, there are additional life-threatening fast-flow blood vascular lesions that include AVM and arteriovenous fistulas (2–4). Lymphatic vascular abnormalities including abnormal lymphatic flow, chylothorax, chylous ascites, and lymphedema have also been described in some patients with CM-AVM (2, 3, 5–8).

CM-AVM1 and CM-AVM2 represent 2 forms of CM-AVM that are caused by mutations in different genes. CM-AVM1, which accounts for approximately 50% of cases, is caused by mutations in the *RASA1* gene that encodes the RASA1 protein, also known as p120 Ras GTPase-activating protein (p120 RasGAP) (1–3). In growth factor receptor (GFR) signaling pathways, RASA1 interacts with the active, GTP-bound form of the Ras small GTP-binding protein (9, 10). This interaction increases the ability of Ras to hydrolyze bound GTP to GDP by several orders of magnitude, resulting in the conversion of Ras to its inactive, GDP-bound state. As such, RASA1 acts as a negative regulator of GFR-induced Ras activation and downstream signaling pathways, such as the mitogen-activated protein kinase (MAPK) pathway that couples cell surface GFR ligand recognition events to cellular outcomes (9, 10).

The vast majority of inherited *RASA1* mutations in CM-AVM1 are nonsense mutations, frameshift mutations, or splice substitutions that result in premature translation termination codons (2, 3). Thus, mutations are thought to be inactivating as transcripts are likely rapidly degraded by nonsense-mediated RNA decay. However, inheritance of 1 null *RASA1* allele is considered insufficient for lesion development. Instead, development of lesions requires the acquisition of an additional somatic second hit mutation in the wild-type *RASA1* allele in endothelial cells (ECs) during development (11, 12). This second hit mutation, together with the germline *RASA1* mutation, renders RASA1-null ECs that are thought to give rise to lesions.

CM-AVM2 accounts for approximately 30% of CM-AVM cases and is phenotypically similar to CM-AVM1 with the addition of telangiectasias (4). The affected gene in CM-AVM2 is *EPHB4*, which encodes the GFR Ephrin receptor B4 (EPHB4). Approximately 50% of *EPHB4* mutations are predicted to result in null alleles as a result of nonsense-mediated RNA decay. The remaining *EPHB4* mutations are missense mutations located mostly in codons that encode amino acids contained in the extracellular domain or the intracellular protein tyrosine kinase (PTK) domain (4). Although not yet demonstrated, it is likely that development of vascular lesions in CM-AVM2 is also dependent upon acquisition of somatic second hit mutations in ECs during development, in this instance in the inherited wild-type *EPHB4* allele.

The occurrence of both *RASA1* and *EPHB4* mutations in CM-AVM suggests that RASA1 and EPHB4 function in the same signaling pathway to regulate vascular development. Consistent with this is the finding that global knockout mice that constitutively lack RASA1, EPHB4, or the EPHB4 ligand, Ephrin B2, all die in midgestation as a consequence of failed vascular development (13–15). Specifically, primitive vascular plexuses formed from vasculogenesis fail to become remodeled through developmental angiogenesis into hierarchical arterial-capillary-venous networks. Studies of conditional RASA1- and EPHB4-deficient mice are also consistent with the notion of a close functional relationship between RASA1 and EPHB4 in the vasculature. Thus, vasculature-specific disruption of *Rasa1* and *Ephb4* blocks the development of venous valves, lymphatic vessel (LV) valves, and lymphovenous valves (16–20).

In its role as a GFR, EPHB4 activates the Ras/MAPK signaling pathway (21). In contrast, RASA1 inhibits Ras/MAPK signaling (10). Therefore, the finding that disruption of *Ephb4* and *Rasa1* in the vasculature in mice results in the same phenotype seems counterintuitive, at least when considering that they function in the same molecular signaling pathway in ECs. However, although EPHB4 can promote Ras/MAPK signaling in some cell types, in human umbilical vein endothelial cells (HUVECs), EPHB4 functions as an inhibitor of Ras/MAPK signaling initiated through other GFRs, such as vascular endothelial growth factor receptor (VEGFR) and Tie2 (21, 22). Moreover, an ability of EPHB4 to dampen Ras/MAPK signaling through these other GFRs is dependent upon expression of RASA1 in HUVECs (21). During EPHB4 signal transduction, RASA1 interacts physically with EPHB4 (22–24). Ephrin B2 binding to EPHB4 induces the kinase activity of EPHB4, resulting in the phosphorylation of multiple EPHB4 tyrosine residues, including Y590 and Y596 in the juxtamembrane (JM) region of the intracellular domain. Phosphorylated Y590 and Y596 are then recognized by 2 Src homology 2 (SH2) domains contained in RASA1, which allow binding of RASA1 to EPHB4 (23, 24). Based on these findings, one straightforward model to account for the role of EPHB4 and RASA1 in vascular development is that in ECs, EPHB4 functions as an inhibitory receptor that serves primarily to recruit RASA1 to the plasma membrane, allowing its juxtaposition to Ras-GTP, an event necessary for Ras inactivation. To test this model, Kawasaki et al. generated zebrafish that expressed a Y590F/Y596F double point mutant of EPHB4 that is unable to bind RASA1 (24). These zebrafish demonstrated the same defect in vascularization of the caudal end of the tail as observed in zebrafish that lack EPHB4 or RASA1 completely. However, it is of note that phosphorylation of these tyrosine residues in other members of the Ephrin receptor family (EPHB2 and EPHA2) is required to switch the kinase domain from a restrained, inactive conformation to an open, active conformation with full kinase activity (25–27). Therefore, it is not possible to conclude from these studies that a physical association between RASA1 and EPHB4 underlies any apparent functional relationship in vascular development.

In a recent study, we investigated the mechanism by which loss of RASA1 in mouse embryos impairs developmental angiogenesis (28). We determined that RASA1-deficient embryonic ECs fail to export an extracellular matrix protein, collagen IV, for deposition in nascent vascular basement membranes. Consequently, ECs undergo apoptosis, either because of an inability to attach to the basement membrane or because of ER stress. Available data are consistent with a model in which dysregulated Ras/MAPK signaling in RASA1-deficient embryonic ECs results in an increased abundance of proline and lysine hydroxylases within the EC ER. The increased abundance of these enzymes results in excessive hydroxylation of collagen IV monomers that impairs their folding and assembly into trimeric collagen IV protomers that are normally exported from the ER via the coat polymer II secretory mechanism. Consequently, drugs that inhibit MAPK signaling, promote collagen IV folding in the ER, or inhibit the activity of collagen proline and lysine hydroxylases, can each rescue the developmental angiogenesis defect in EC-specific RASA1-deficient embryos (28). Although required for developmental angiogenesis in the embryo, RASA1 is not required for the maintenance of the blood vasculature in adults (16, 29). However, RASA1 is required for retinal angiogenesis in neonates and pathological angiogenesis to solid tumors in adults (28). These findings are consistent with a requirement for de novo EC synthesis of collagen IV in these responses.

To obtain further evidence that EPHB4 and RASA1 function in the same signaling pathway to regulate the blood vasculature, we examined the basis of a requirement for EPHB4 for developmental angiogenesis in mice. We report that loss of EPHB4 in ECs during developmental angiogenesis resulted in their inability to export collagen IV from the EC ER, which led to their apoptotic death. We also show that like RASA1, EPHB4 was necessary for retinal angiogenesis in newborns and pathological angiogenesis in adults. However, as revealed with the use of a potentially novel EPHB4-knockin model, physical association of EPHB4 with RASA1 was not required for EPHB4 to promote any of these angiogenic responses.

## Results

### Disruption of Ephb4 during developmental angiogenesis results in apoptotic death of ECs.

To understand the role of EPHB4 in developmental angiogenesis, mice with a conditional allele of *Ephb4* in which exons 2 and 3 were flanked by *loxP* sites (*Ephb4^fl^*) were crossed with ubiquitin promoter-driven ert2cre (*Ub^ert2cre^*) transgenic mice to generate *Ephb4^fl/fl^* *Ub^ert2cre^* and control *Ephb4^fl/fl^* cre-negative littermate embryos. Pregnant dams carrying embryos of both genotypes were administered tamoxifen (TM) at E13.5 and embryos were harvested at E18.5. By E13.5 vasculogenesis is complete and from E13.5 to E18.5 developmental angiogenesis predominates (30). When examined at E18.5, cre-positive embryos exhibited severe cutaneous hemorrhage that manifested visibly across most of the surface of the embryo (Table 1 and Supplemental Figure 1A; supplemental material available online with this article; https://doi.org/10.1172/jci.insight.156928DS1). Staining of tissue sections with H&E and anti-CD31 and anti–lymphatic vessel endothelial hyaluronan receptor 1 (anti–LYVE-1) antibodies confirmed hemorrhage that was associated with damaged blood vessels (BVs) and a near absence of LVs at this time point consistent with an edematous appearance of embryos (Supplemental Figure 1, A and B). The same hemorrhagic and edematous phenotype was observed in TM-treated *Ephb4^fl/fl^* *Ub^ert2cre^* embryos that carried distinct *Ephb4^fl^* alleles in which exon 1 was flanked by *loxP* sites (Supplemental Figure 2, A and B, and Table 1). To determine if hemorrhage was associated with the apoptotic death of ECs, tissue sections from *Ephb4^fl/fl^* *Ub^ert2cre^* embryos were stained with antibodies that detect the activated form of caspase 3. In both strains of *Ephb4^fl/fl^* *Ub^ert2cre^* mice, apoptotic ECs were readily identified in BVs of skin (Supplemental Figure 1, C and D; and Supplemental Figure 2, C and D). In all subsequent studies with *Ephb4^fl^* mice, we used the exon 2–3 *loxP*-flanked allele.

**Figure 1:**
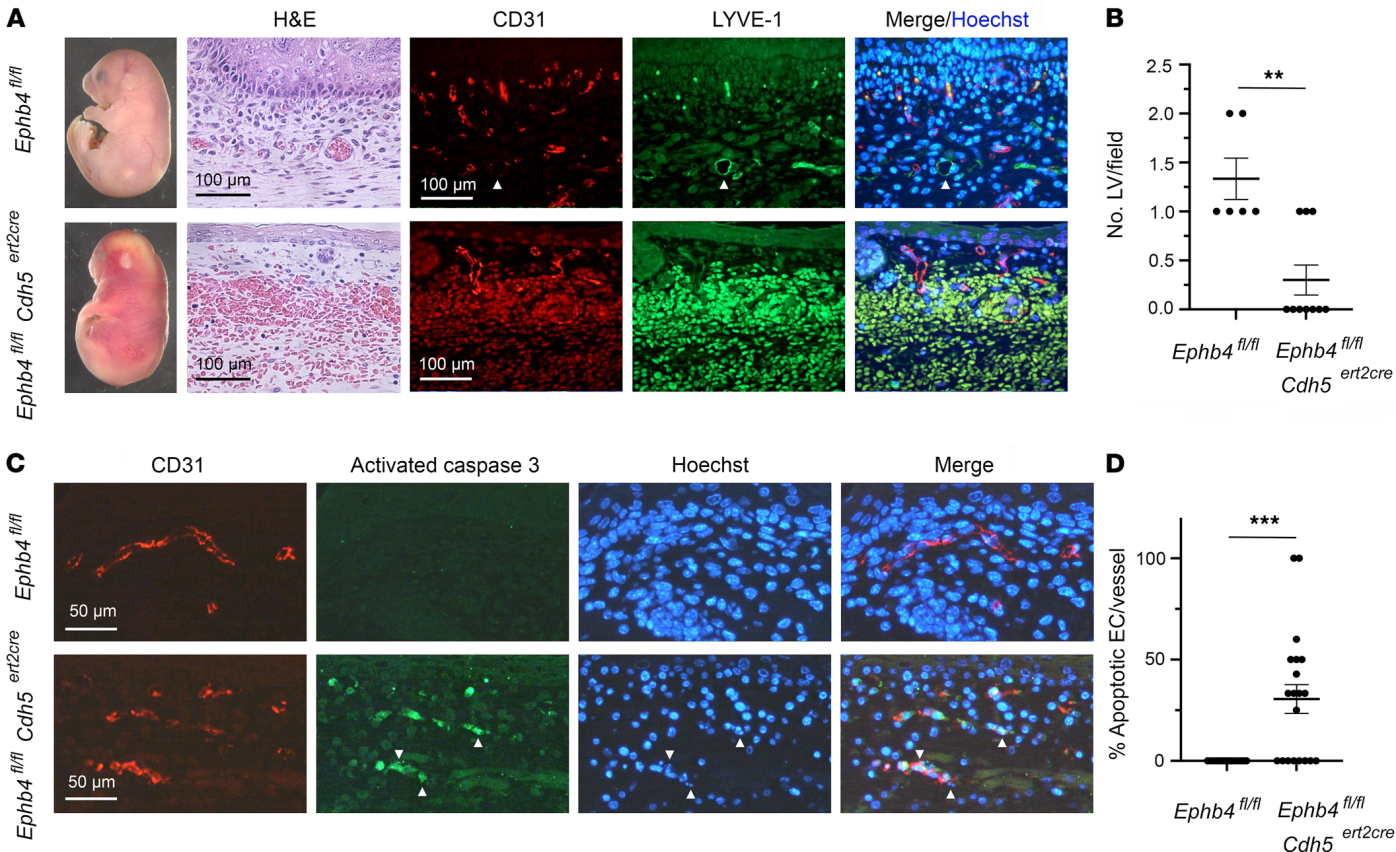
Hemorrhage, EC apoptosis, and paucity of LVs following induced EC-specific disruption of *Ephb4* during developmental angiogenesis. TM was administered to *Ephb4^fl/fl^* and *Ephb4^fl/fl^* *Cdh5^ert2cre^* embryos at E13.5 and embryos were harvested at E18.5. (**A**) Images at left show extensive cutaneous hemorrhage and an edematous appearance of *Ephb4^fl/fl^* *Cdh5^ert2cre^* embryos. H&E staining of skin sections demonstrated vascular hemorrhage, and combined anti-CD31 and anti–LYVE-1 antibody staining of sections revealed a reduced number of intact CD31^lo^LYVE-1^+^ initial LVs in *Ephb4^fl/fl^* *Cdh5^ert2cre^* embryos (shown with arrowheads in images of *Ephb4^fl/fl^* sections). (**B**) Plot shows the number of identified CD31^lo^LYVE-1^+^ LVs in randomly selected 200 × 200 μm areas of skin. Bars show the mean ± 1 SEM of LVs/field (*Ephb4^fl/fl^*, *n* = 5; *Ephb4^fl/fl^* *Cdh5^ert2cre^*, *n* = 10). (**C**) Skin sections were stained with anti-CD31 and anti–activated caspase 3 antibodies and Hoechst to identify apoptotic ECs. Examples of activated caspase 3–positive ECs with fragmented nuclei in images of *Ephb4^fl/fl^* *Cdh5^ert2cre^* sections are indicated with arrowheads. (**D**) Plot shows the percentage of apoptotic ECs in individual CD31^+^ BVs in skin selected from multiple randomly chosen areas. Bars show the mean ± 1 SEM of percentage apoptotic ECs per vessel (*Ephb4^fl/fl^*, *n* = 14; *Ephb4^fl/fl^* *Cdh5^ert2cre^*, *n* = 20). **, *P* < 0.01; ***, *P* < 0.001; Mann-Whitney test.

We next examined if EC apoptosis and hemorrhage resulted from loss of EPHB4 within ECs. For this purpose, we generated *Ephb4^fl/fl^* embryos with an EC-specific *Cdh5-ert2cre* driver. Administration of TM to pregnant dams at E13.5 resulted in the same cutaneous hemorrhagic phenotype and near absence of LVs associated with apoptotic death of ECs when examined at E18.5 (Figure 1 and Table 1). Therefore, embryonic vascular phenotypes observed upon induced global loss of EPHB4 are consequent to loss of EPHB4 in ECs specifically.

### Loss of EPHB4 in ECs during developmental angiogenesis results in accumulation of collagen IV within the EC ER.

**Figure 2:**
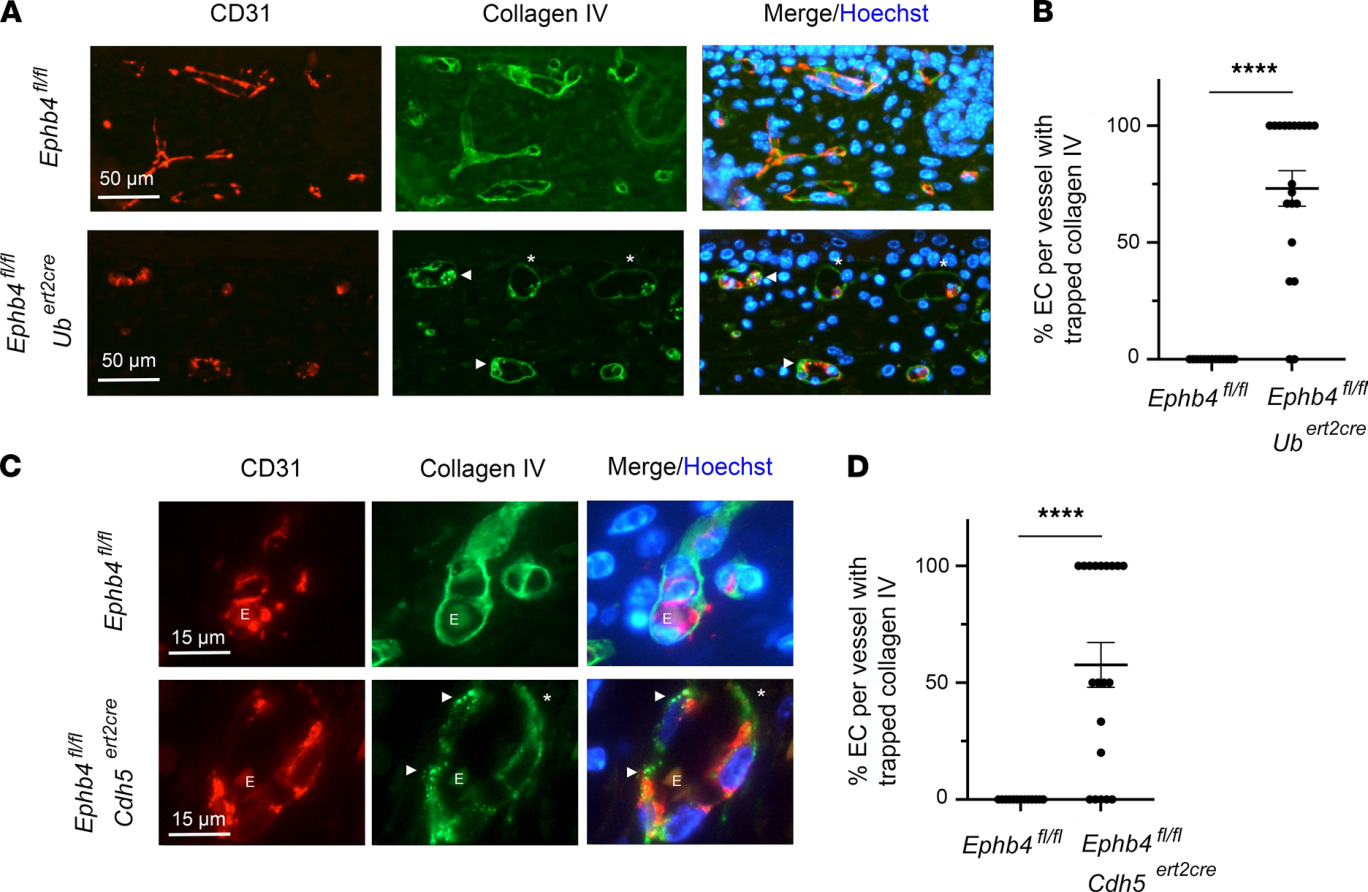
Collagen IV accumulation in ECs of induced EPHB4-deficient embryos. TM was administered to *Ephb4^fl/fl^* *Ub^ert2cre^*, *Ephb4^fl/fl^* *Cdh5^ert2cre^*, and corresponding littermate *Ephb4^fl/fl^* embryos at E13.5. (**A** and **C**) Embryos were harvested at E18.5 and skin sections were stained with anti-CD31 and anti–collagen IV antibodies and Hoechst. Note intracellular accumulation of collagen IV in ECs of *Ephb4^fl/fl^* *Ub^ert2cre^* embryos (**A**) and *Ephb4^fl/fl^* *Cdh5^ert2cre^* embryos (**C**) (examples highlighted with arrowheads) and relative paucity of collagen IV in basement membranes (asterisks). E, erythrocyte. (**B** and **D**) Plots show the percentage of EC with intracellular collagen IV puncta in individual CD31^+^ BV in skin of embryos selected from multiple randomly chosen areas. Bars show the mean ± 1 SEM of percentage EC with collagen IV accumulation (**B**, *Ephb4^fl/fl^*, *n* = 13; *Ephb4^fl/fl^* *Ub^ert2cre^*, *n* = 20), (**C**, *Ephb4^fl/fl^*, *n* = 13; *Ephb4^fl/fl^* *Cdh5^ert2cre^*, *n* = 20). ****, *P* < 0.0001; Mann-Whitney test.

**Figure 3:**
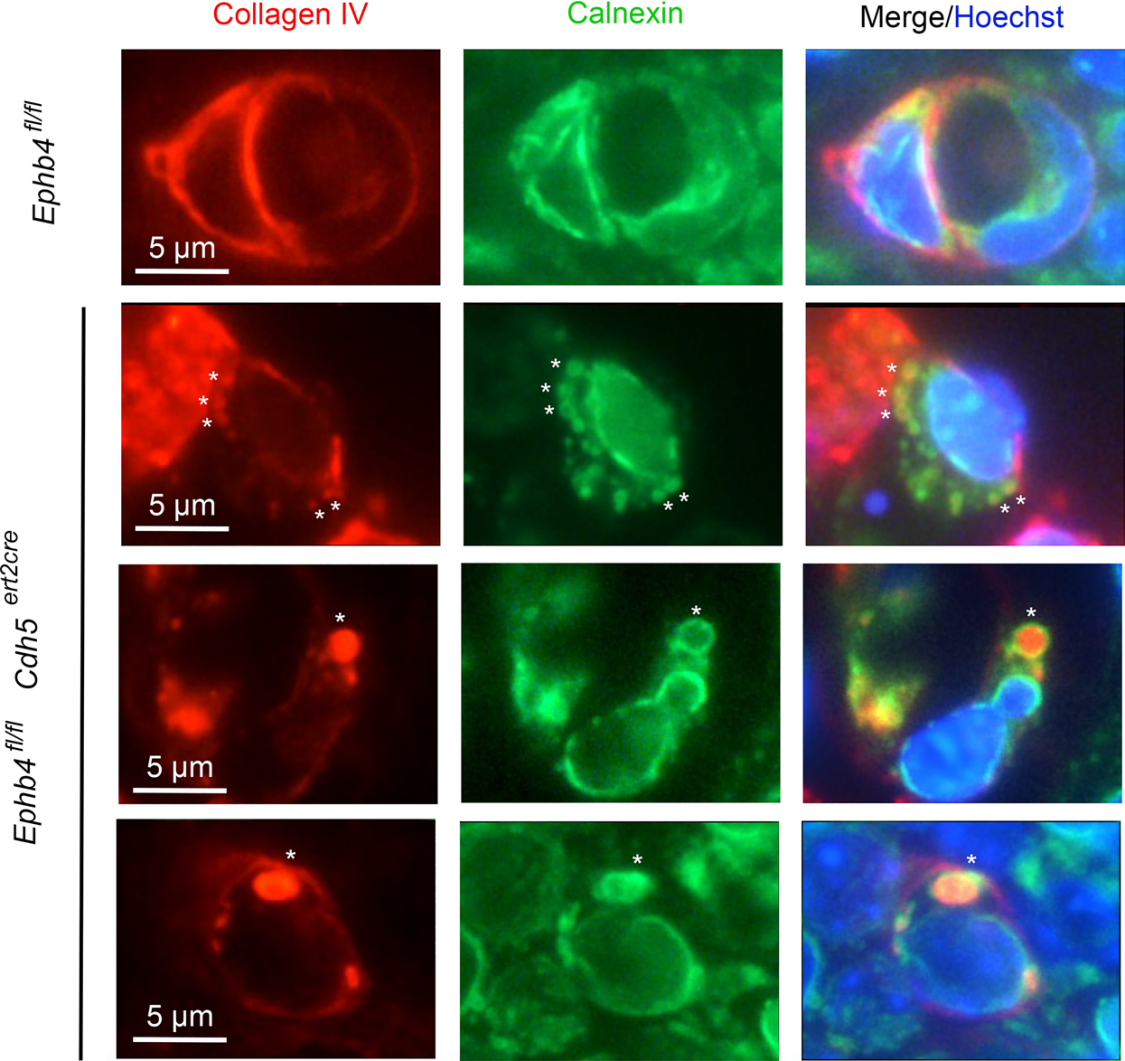
Collagen IV is retained within the ER of induced EPHB4-deficient ECs during developmental angiogenesis. TM was administered to *Ephb4^fl/fl^* and *Ephb4^fl/fl^* *Cdh5^ert2cre^* embryos at E13.5 and embryos were harvested at E18.5. Skin sections were stained with anti–collagen IV antibodies and antibodies against calnexin to identify the ER. Representative images of individual BVs are shown. Note collagen IV puncta surrounded by rings of calnexin in *Ephb4^fl/fl^* *Cdh5^ert2cre^* embryos (asterisks).

Apoptotic death of induced RASA1-deficient ECs during developmental angiogenesis is secondary to the inability of ECs to export collagen IV for deposition in vascular basement membranes (28). To investigate if induced loss of EPHB4 in ECs during developmental angiogenesis also resulted in intracellular accumulation of collagen IV in ECs, tissue sections from E18.5 *Ephb4^fl/fl^* *Ub^ert2cre^* and *Ephb4^fl/fl^* *Cdh5^ert2cre^* embryos administered TM at E13.5 were stained with anti–collagen IV antibodies. Intracellular accumulation of collagen IV was readily apparent in ECs of skin of *Ephb4^fl/fl^* *Ub^ert2cre^* and *Ephb4^fl/fl^* *Cdh5^ert2cre^* embryos but not in ECs of corresponding littermate cre-negative embryos (Figure 2, A–D). Similarly, intracellular accumulation of collagen IV was observed in ECs of TM-treated exon 1 *loxP-*flanked *Ephb4^fl/fl^* *Ub^ert2cre^* embryos (Supplemental Figure 3). To determine the subcellular location of the intracellular collagen IV, sections were additionally stained with antibodies to identify different cell organelles. Within ECs of *Ephb4^fl/fl^* *Cdh5^ert2cre^* embryos, puncta of collagen IV were identified that were surrounded by rings of calnexin, a transmembrane-resident ER protein (Figure 3). In addition, colocalization of collagen IV puncta with calreticulin, a lumenal ER protein, was observed (Supplemental Figure 4). In contrast, no colocalization of collagen IV with markers that identify the ER-Golgi intermediate compartment (LMNA1), the Golgi (TGN46), or lysosomes (LAMP-1) was apparent (Supplemental Figure 4). Thus, loss of EPHB4 in ECs during developmental angiogenesis leads to the accumulation of collagen IV within the EC ER.

### Partial rescue of vascular phenotypes in induced EPHB4-deficient embryos by 4PBA.

**Figure 4:**
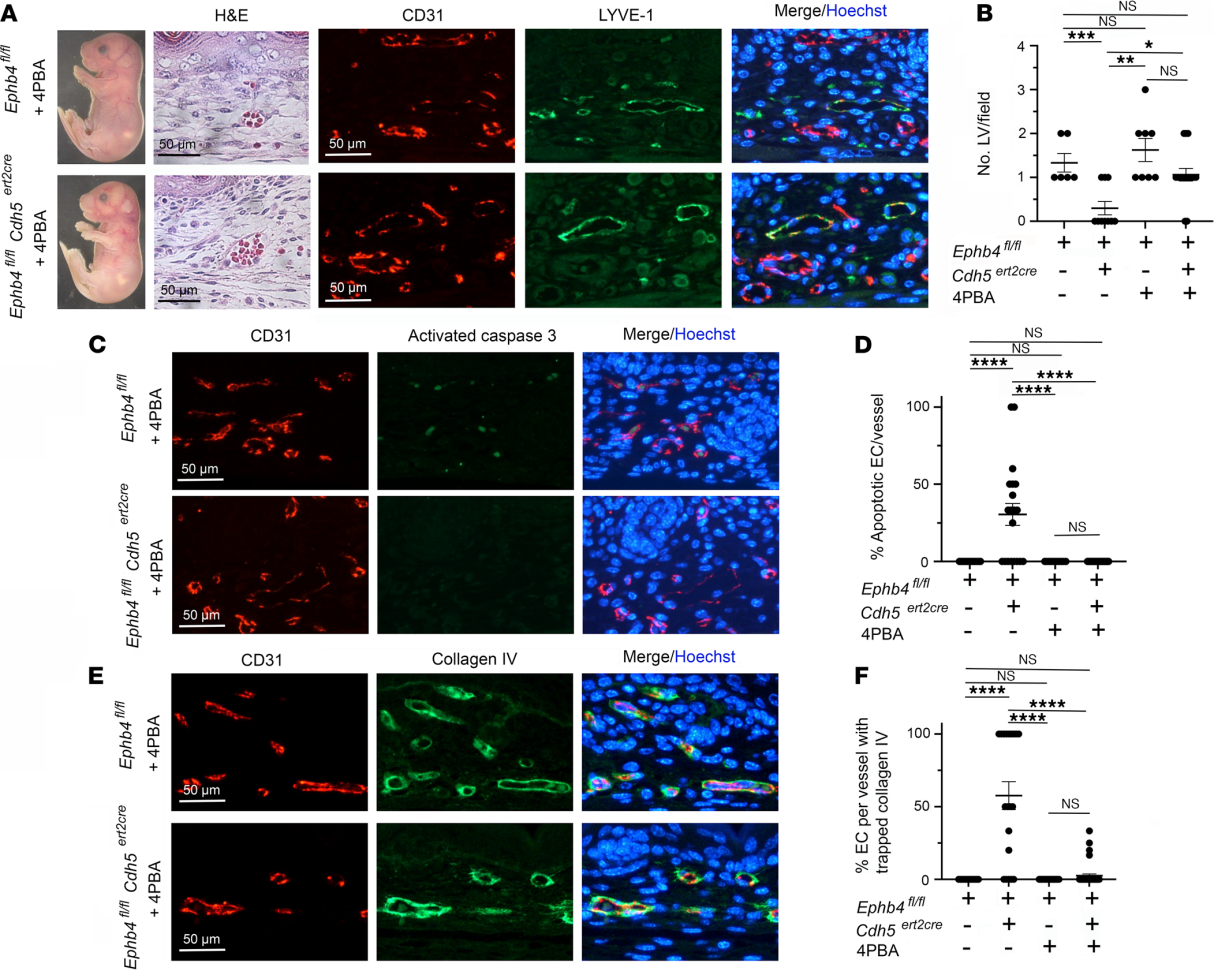
Partial rescue of developmental angiogenesis in induced EPHB4-deficient mice by 4PBA. TM and 4PBA were administered to *Ephb4^fl/fl^* and *Ephb4^fl/fl^* *Cdh5^ert2cre^* embryos at E13.5, and embryos were harvested at E18.5. (**A**) Shown are an *Ephb4^fl/fl^* embryo and an *Ephb4f^l/fl^ Cdh5^ert2cre^* embryo with mild cutaneous hemorrhage (see Table 1) and intact LVs confirmed by staining of skin sections with H&E and anti-CD31 and anti–LYVE-1 antibodies. (**B**) Plot shows the mean ± 1 SEM of CD31^lo^LYVE-1^+^ LVs/field in randomly selected 200 x 200 μm areas of skin of *Ephb4^fl/fl^* embryos and *Ephb4^fl/fl^* *Cdh5^ert2cre^* embryos with mild hemorrhage (*Ephb4^fl/fl^* TM + 4PBA, *n* = 8; *Ephb4^fl/fl^* *Cdh5^ert2cre^* TM + 4PBA, *n* = 16). Data from *Ephb4^fl/fl^* and *Ephb4^fl/fl^* *Cdh5^ert2cre^* embryos treated with TM alone are also shown (Figure 1). (**C**) Skin sections were stained with anti-CD31 and anti–activated caspase 3 antibodies and Hoechst to identify apoptotic ECs. (**D**) Plot shows the mean ± 1 SEM of the percentage apoptotic ECs in individual CD31^+^ BVs from randomly chosen areas (*Ephb4^fl/fl^ Cdh5^ert2cre^* embryos with mild hemorrhage) (*Ephb4^fl/fl^* TM + 4PBA, *n* = 11; *Ephb4^fl/fl^* *Cdh5^ert2cre^* TM + 4PBA, *n* = 42). (**E**) Skin sections were stained with anti-CD31 and anti–collagen IV antibodies and Hoechst to determine the distribution of collagen IV in BVs. (**F**) Plot shows the mean ± 1 SEM of percentage ECs with collagen accumulation in individual CD31^+^ BVs from randomly chosen areas (*Ephb4^fl/fl^ Cdh5^ert2cre^* embryos with mild hemorrhage) (*Ephb4^fl/fl^* TM + 4PBA, *n* = 19; *Ephb4^fl/fl^* *Cdh5^ert2cre^* TM + 4PBA, *n* = 38). *, *P* < 0.05; **, *P* < 0.01; ***, *P* < 0.001; ****, *P* < 0.0001; 1-way ANOVA with Tukey.

In induced RASA1-deficient embryos, collagen IV is retained within the EC ER because it is improperly folded. Evidence for this is derived from the observation that the small molecular chaperone 4PBA, which promotes collagen IV folding, rescues EC collagen IV export, EC apoptosis, and hemorrhage in induced RASA1-deficient embryos (28, 31, 32). To examine if 4PBA could also rescue vascular phenotypes in induced EPHB4-deficient embryos, 4PBA was administered to *Ephb4^fl/fl^* and *Ephb4^fl/fl^* *Cdh5^ert2cre^* embryos at the same time as TM at E13.5 and for each day of development thereafter until embryo harvest at E18.5. Cotreatment of embryos with 4PBA partially rescued hemorrhage as evidenced by the absence of hemorrhage or much-reduced hemorrhage (mild hemorrhage) in most embryos (Figure 4 and Table 1). Immunostaining of skin sections of embryos with mild hemorrhage revealed mostly intact BVs and a normal number of LVs (Figure 4, A and B). Apoptotic ECs could not be identified in BVs of these embryos, and collagen IV export was mostly normal (Figure 4, C–F). These findings are consistent with the notion that impaired folding of collagen IV in EPHB4-deficient ECs during developmental angiogenesis is responsible for EC apoptosis, reduced LV density, and hemorrhage.

### Partial rescue of vascular phenotypes in induced EPHB4-deficient embryos by 2,4PDCA.

**Figure 5:**
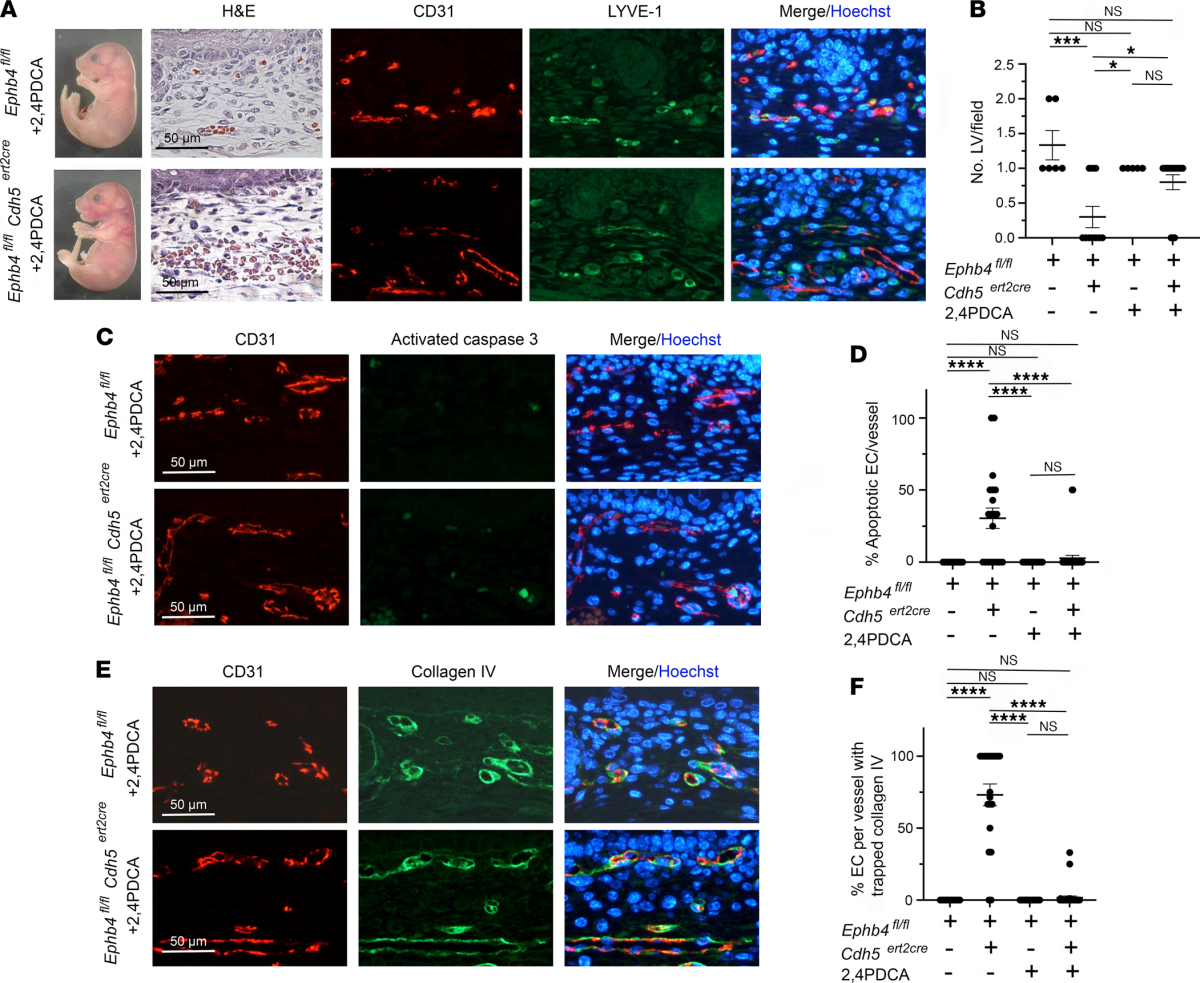
Partial rescue of developmental angiogenesis in induced EPHB4-deficient mice by 2,4PDCA. TM and 2,4PDCA were administered to *Ephb4^fl/fl^* and *Ephb4^fl/fl^* *Cdh5^ert2cre^* embryos at E13.5, and embryos were harvested at E18.5. (**A**) Shown are an *Ephb4^fl/fl^* embryo and an *Ephb4f^l/fl^ Cdh5^ert2cre^* embryo with mild cutaneous hemorrhage (see Table 1) and intact LVs confirmed by staining of skin sections with H&E and anti-CD31 and anti–LYVE-1 antibodies. (**B**) Plot shows the mean ± 1 SEM of CD31^lo^LYVE-1^+^ LVs/field in randomly selected 200 x 200 μm areas of skin of *Ephb4^fl/fl^* embryos and *Ephb4^fl/fl^* *Cdh5^ert2cre^* embryos with mild hemorrhage (*Ephb4^fl/fl^* TM + 2,4PDCA, *n* = 5; *Ephb4^fl/fl^ Cdh5^ert2cre^* TM + 2,4PDCA, *n* = 15). Data from *Ephb4^fl/fl^* and *Ephb4^fl/fl^* *Cdh5^ert2cre^* embryos treated with TM alone are also shown (Figure 1). (**C**) Skin sections were stained with anti-CD31 and anti–activated caspase 3 antibodies and Hoechst to identify apoptotic ECs. (**D**) Plot shows the mean ± 1 SEM of percentage apoptotic ECs in individual CD31^+^ BVs from randomly chosen areas (*Ephb4^fl/fl^ Cdh5^ert2cre^* embryos with mild hemorrhage) (*Ephb4^fl/fl^* TM + 2,4PDCA, *n* = 16; *Ephb4^fl/fl^* *Cdh5^ert2cre^* TM + 2,4PDCA, *n* = 36). (**E**) Skin sections were stained with anti-CD31 and anti–collagen IV antibodies and Hoechst to determine the distribution of collagen IV in BVs. (**F**) Plot shows the mean ± 1 SEM of percentage of ECs with collagen accumulation in individual CD31^+^ BVs from randomly chosen areas (*Ephb4^fl/fl^ Cdh5^ert2cre^* embryos with mild hemorrhage) (*Ephb4^fl/fl^* TM + 2,4PDCA, *n* = 19; *Ephb4^fl/fl^* *Cdh5^ert2cre^* TM + 2,4PDCA, *n* = 36). *, *P* < 0.05; ***, *P* < 0.001; ****, *P* < 0.0001; 1-way ANOVA with Tukey.

Induced loss of RASA1 during developmental angiogenesis results in an increased abundance of collagen IV proline and lysine hydroxylases in ECs (28). These enzymes belong to a family of enzymes known as 2-oxoglutarate–dependent (2OG-dependent) oxygenases (33). The increased abundance of hydroxylases is thought to result in overhydroxylation of collagen IV on prolines and lysines that could affect proper folding. Consistent with this possibility is the finding that drugs that inhibit 2OG-dependent oxygenases also rescue EC collagen IV export, EC apoptosis, and hemorrhage resulting from loss of RASA1 during developmental angiogenesis (28). To examine if inhibition of 2OG-dependent oxygenases could also rescue vascular phenotypes in induced EPHB4-deficient embryos, we tested the effect of the 2OG-dependent oxygenase inhibitor, 2,4PDCA (33). Similar to 4PBA treatment, administration of 2,4PDCA to *Ephb4^fl/fl^* *Cdh5^ert2cre^* embryos at the same time as TM at E13.5 and for all subsequent days up to embryo harvest at E18.5 partially rescued EC collagen IV export, EC apoptosis, and hemorrhage (Figure 5 and Table 1). This finding provides evidence of a role for 2OG-dependent oxygenases in collagen IV misfolding in EPHB4-deficient ECs and is further supportive of a functional link between RASA1 and EPHB4 in the regulation of vascular development.

### EPHB4 functions as a negative regulator of Ras/MAPK signaling in ECs, and an inhibitor of Ras/MAPK signaling ameliorates blood vascular phenotypes resulting from loss of EPHB4 during developmental angiogenesis.

**Figure 6:**
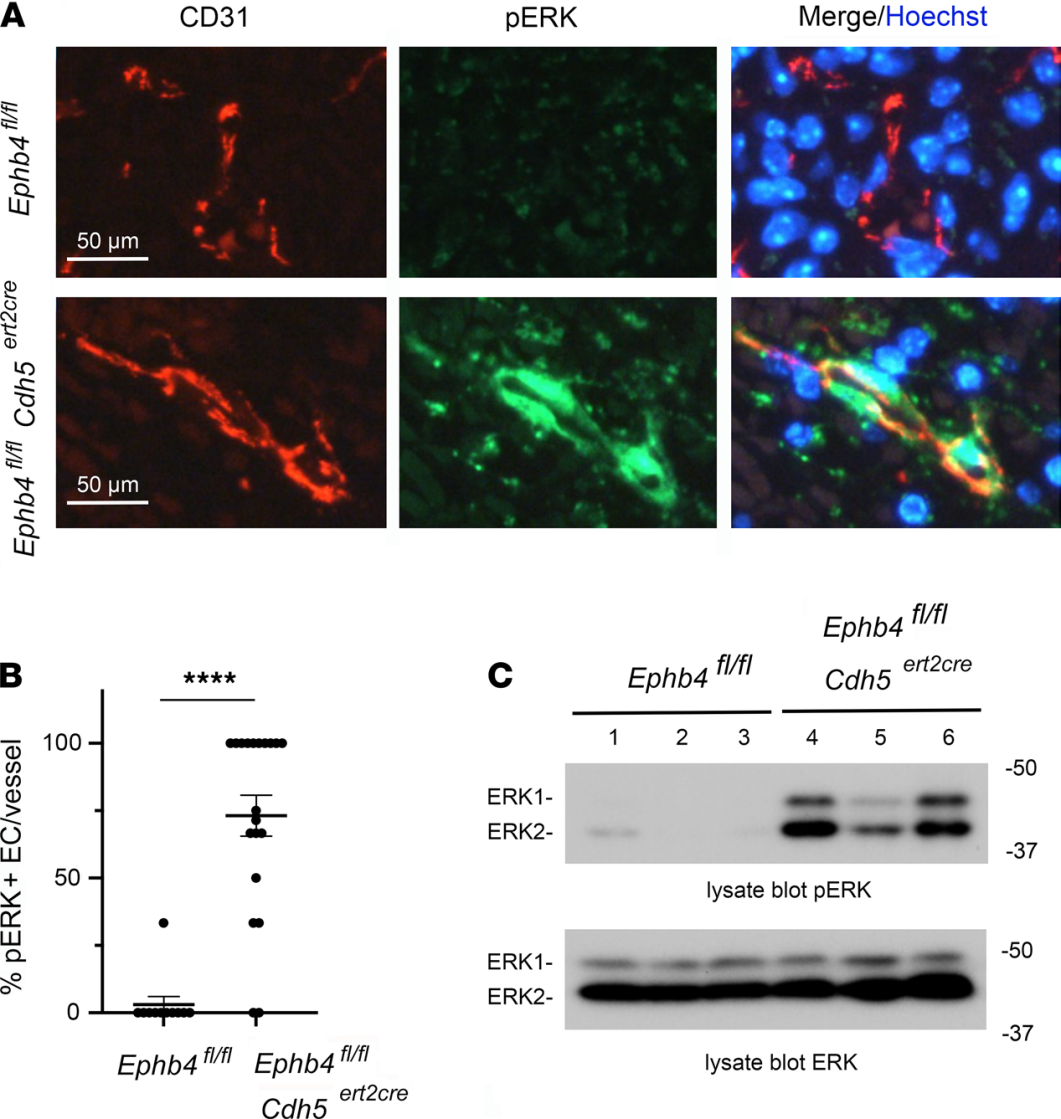
Activation of MAPK in EPHB4-deficient ECs during developmental angiogenesis. (**A**) TM was administered to *Ephb4^fl/fl^* and *Ephb4^fl/fl^* *Cdh5^ert2cre^* embryos at E13.5 and embryos were harvested at E18.5. (**A**) Skin sections were stained with anti-CD31 and anti–phospho-ERK MAPK antibodies (pERK) and Hoechst. Note strong activation of MAPK in ECs of *Ephb4^fl/fl^* *Cdh5^ert2cre^* embryos. (**B**) Plot shows the percentage of pERK^+^ ECs per BV identified in randomly selected areas of skin. Bars show the mean ± 1 SEM of percentage pERK^+^ ECs per vessel (*Ephb4^fl/fl^*, *n* = 11; *Ephb4^fl/fl^* *Cdh5^ert2cre^*, *n* = 20). ****, *P* < 0.0001, Mann-Whitney test. (**C**) Liver tissue from individual embryos was analyzed by Western blotting using pERK antibodies. Note constitutive activation of MAPK in *Ephb4^fl/fl^* *Cdh5^ert2cre^* liver samples. See complete unedited blots in the supplemental material.

Since loss of EPHB4 and RASA1 during developmental angiogenesis resulted in the same vascular phenotypes, we next asked if EPHB4, like RASA1, functions as a negative regulator of Ras/MAPK signaling in ECs in vivo, as has been reported in vitro (21, 22). To examine this, skin sections from *Ephb4^fl/fl^* and *Ephb4^fl/fl^* *Cdh5^ert2cre^* E15.5 embryos treated with TM on E13.5 were stained with antibodies against phosphorylated activated forms of MAPK. Constitutive activation of MAPK was observed in ECs in the majority of skin BVs in *Ephb4^fl/fl^* *Cdh5^ert2cre^* E15.5 embryos at E18.5, whereas very little MAPK activation was observed in ECs in skin of *Ephb4^fl/fl^* embryos at this time (Figure 6, A and B). To confirm this finding, we examined MAPK activation by Western blotting of liver lysates of E18.5 *Ephb4^fl/fl^* and *Ephb4^fl/fl^* *Cdh5^ert2cre^* E15.5 embryos treated with TM on E13.5 (Figure 6C). In these experiments, constitutive MAPK activation was consistently observed in the *Ephb4^fl/fl^* *Cdh5^ert2cre^* embryos.

**Figure 7:**
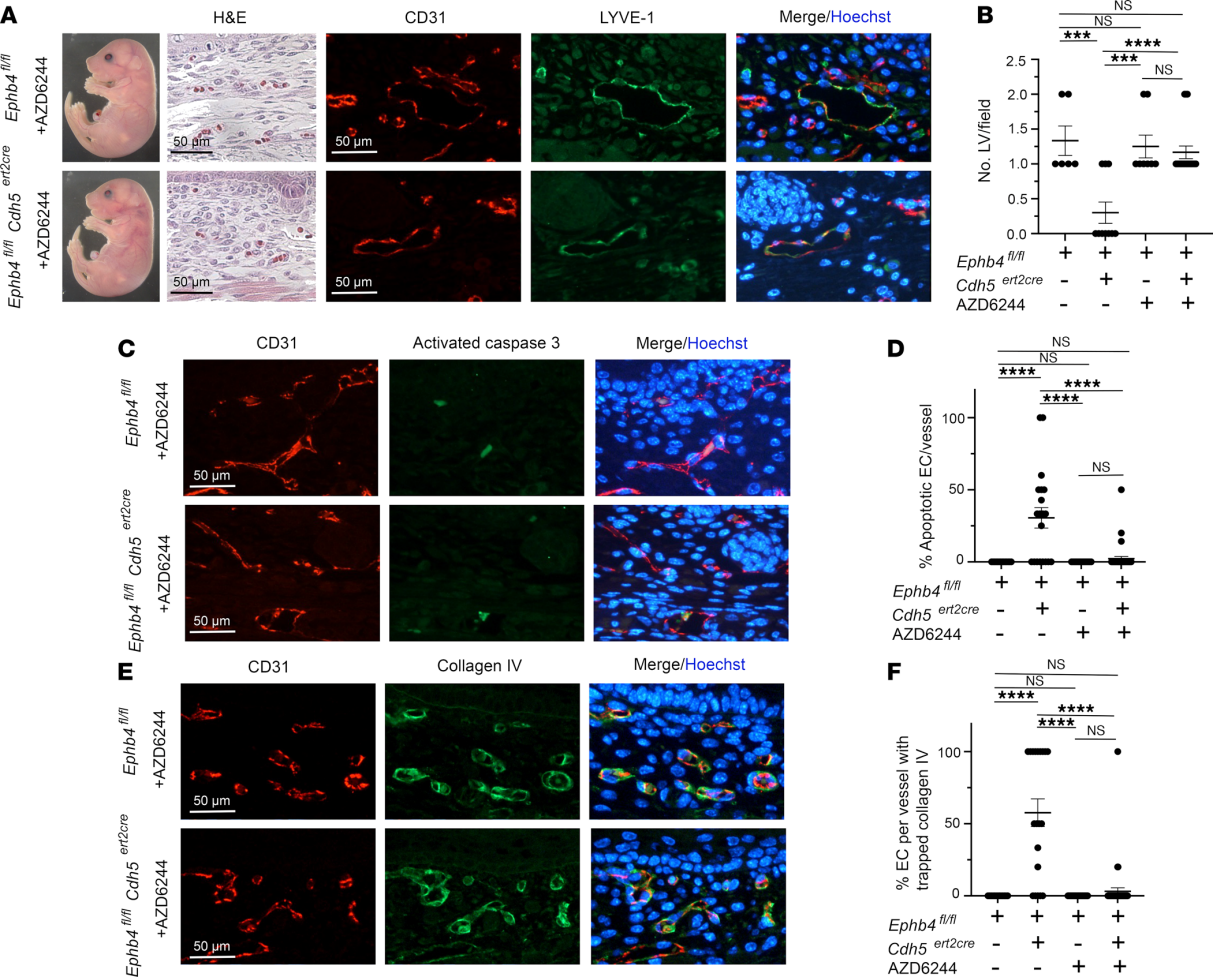
Partial rescue of developmental angiogenesis in induced EPHB4-deficient mice by AZD6244. TM and AZD6244 were administered to *Ephb4^fl/fl^* and *Ephb4^fl/fl^* *Cdh5^ert2cre^* embryos at E13.5, and embryos were harvested at E18.5. (**A**) Shown are an *Ephb4^fl/fl^* embryo and an *Ephb4f^l/fl^ Cdh5^ert2cre^* embryo with mild cutaneous hemorrhage (see Table 1) and intact LVs confirmed by staining of skin sections with H&E and anti-CD31 and anti–LYVE-1 antibodies. (**B**) Plot shows the mean ± 1 SEM of CD31^lo^LYVE-1^+^ LVs/field in randomly selected 200 x 200 μm areas of skin of *Ephb4^fl/fl^* embryos and *Ephb4^fl/fl^* *Cdh5^ert2cre^* embryos with mild hemorrhage (*Ephb4^fl/fl^* TM + AZD6244, *n* = 8; *Ephb4^fl/fl^ Cdh5^ert2cre^* TM + AZD6244, *n* = 18). Data from *Ephb4^fl/fl^* and *Ephb4^fl/fl^* *Cdh5^ert2cre^* embryos treated with TM alone are also shown (Figure 1). (**C**) Skin sections were stained with anti-CD31 and anti–activated caspase 3 antibodies and Hoechst to identify apoptotic ECs. (**D**) Plot shows the mean ± 1 SEM of percentage apoptotic ECs in individual CD31^+^ BVs from randomly chosen areas (*Ephb4^fl/fl^ Cdh5^ert2cre^* embryos with mild hemorrhage) (*Ephb4^fl/fl^* TM + AZD6244, *n* = 16; *Ephb4^fl/fl^* *Cdh5^ert2cre^* TM + AZD6244, *n* = 37). (**E**) Skin sections were stained with anti-CD31 and anti–collagen IV antibodies and Hoechst to determine the distribution of collagen IV in BVs. (**F**) Plot shows the mean ± 1 SEM of percentage of ECs with collagen accumulation in individual CD31^+^ BVs from randomly chosen areas (*Ephb4^fl/fl^ Cdh5^ert2cre^* embryos with mild hemorrhage) (*Ephb4^fl/fl^* TM + 4PBA, *n* = 19; *Ephb4^fl/fl^* *Cdh5^ert2cre^* TM + 4PBA alone, *n* = 44). ***, *P* < 0.001; ****, *P* < 0.0001; 1-way ANOVA with Tukey.

We next asked if inhibition of Ras/MAPK signaling could rescue developmental EC phenotypes following loss of EPHB4. For this purpose, mice were administered an MEK inhibitor, AZD6244, at the same time as TM at E13.5 and on all subsequent days until embryo harvest at E18.5. AZD6244 completely rescued or reduced hemorrhage in most *Ephb4^fl/fl^* *Cdh5^ert2cre^* embryos (Figure 7, A and B, and Table 1). In embryos with reduced hemorrhage, LV density in skin was restored, there were many fewer BVs with apoptotic ECs, and EC export of collagen IV was mostly normal (Figure 7, A–F). These findings are in accord with the notion that dysregulated Ras/MAPK signaling in ECs is responsible for the vascular phenotypes that result from loss of EPHB4 during developmental angiogenesis.

### EPHB4 is required for retinal angiogenesis in newborns.

With few exceptions, induced disruption of *Rasa1* past E15.5 does not result in spontaneous BV abnormalities (16, 18, 29, 34). Collagens are considered some of the most stable proteins in the animal kingdom (35). Hence, a lack of requirement of RASA1 for maintenance of the blood vasculature after E15.5 could reflect a much-reduced need of ECs to continue to engage in high-rate collagen IV synthesis to remain attached to the basement membrane. However, it is expected that different forms of neoangiogenesis, such as retinal angiogenesis in newborns, would require de novo synthesis of collagen IV by ECs for BV growth. Accordingly, we showed previously that RASA1 is required for retinal angiogenesis in newborns (28).

**Figure 8:**
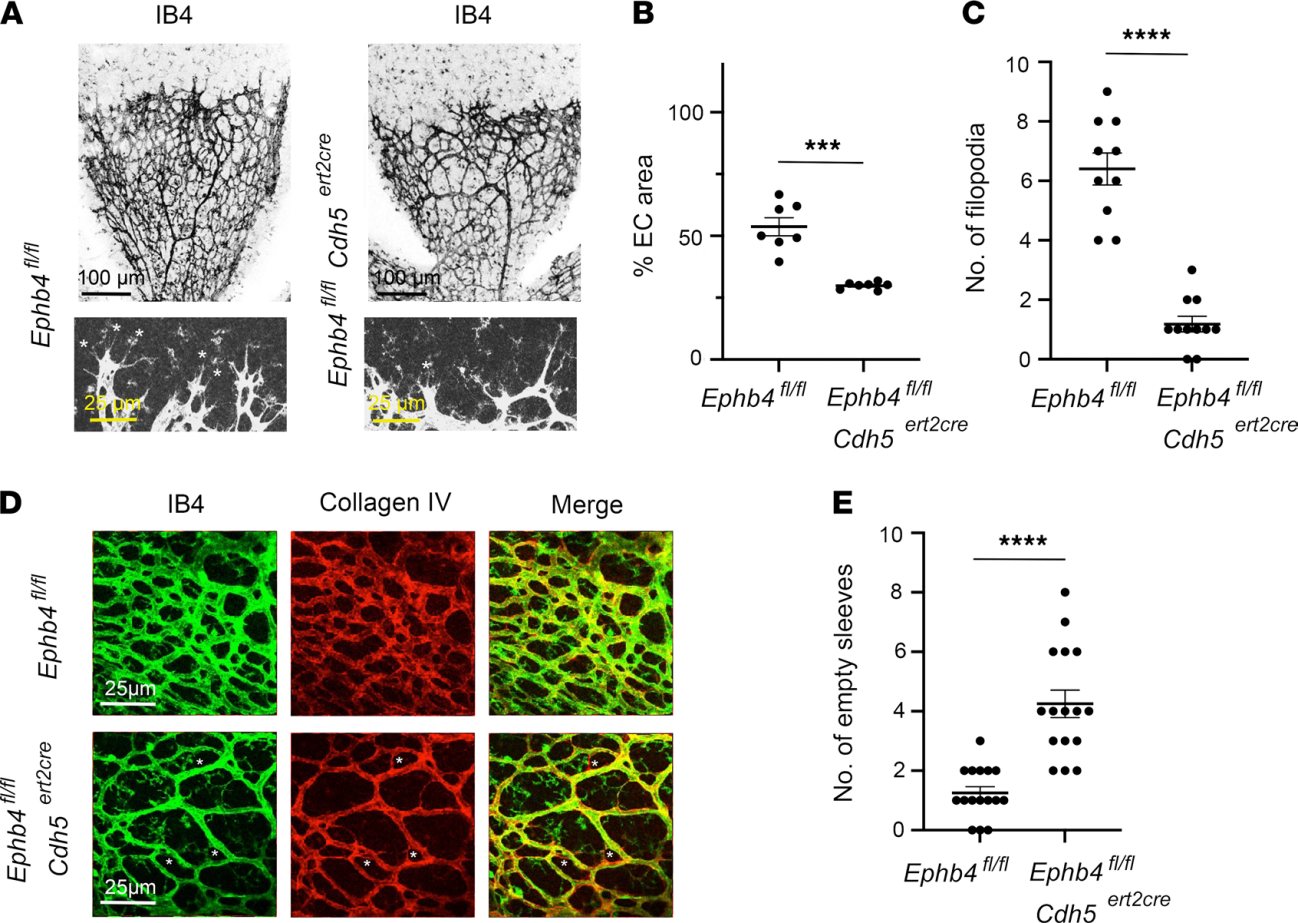
Impaired retinal angiogenesis in induced EC-specific EPHB4-deficient mice. TM was administered to *Ephb4^fl/fl^* and *Ephb4^fl/fl^* *Cdh5^ert2cre^* mice at P1 and P2. Retinas were harvested at P6 and stained with IB4 to identify BVs and anti–collagen IV antibodies. (**A**) Representative low-power images of IB4 staining are shown at top. Representative higher power images of IB4 staining at the angiogenic front are shown at bottom. Asterisks indicate filopodia. (**B**) Plot shows the percentage coverage of retinas with ECs. Bars show the mean ± 1 SEM of percentage EC coverage of individual retinas (*Ephb4^fl/fl^*, *n* = 7; *Ephb4^fl/fl^* *Cdh5^ert2cre^*, *n* = 7). (**C**) Plot shows the number of filopodia per 300 μm of angiogenic front that were randomly selected. Bars show the mean ± 1 SEM of filopodia (*Ephb4^fl/fl^*, *n* = 10; *Ephb4^fl/fl^* *Cdh5^ert2cre^*, *n* = 11). (**D**) Shown are representative images of IB4 and anti–collagen IV staining. Empty collagen sleeves are indicated with asterisks. (**E**) Plot shows the number of empty sleeves in randomly selected 200 μm x 200 μm areas of retinas. Bars show the mean ± 1 SEM of empty sleeves (*Ephb4^fl/fl^*, *n* = 16; *Ephb4^fl/fl^* *Cdh5^ert2cre^*, *n* = 16). ***, *P* < 0.001; ****, *P* < 0.0001; Student’s 2-sample *t* test.

To investigate if EPHB4 is also required for retinal angiogenesis, *Ephb4^fl/fl^* and *Ephb4^fl/fl^* *Cdh5^ert2cre^* mice were administered TM at P1 and retinas were harvested at P6. Retinas were then examined by whole mount staining using isolectin B4 (IB4) to identify BVs. These analyses revealed a reduced density of BVs in retinas from *Ephb4^fl/fl^* *Cdh5^ert2cre^* mice compared with *Ephb4^fl/fl^* controls (Figure 8, A and B). In addition, the number of filopodia at the angiogenic front was reduced in the *Ephb4^fl/fl^* *Cdh5^ert2cre^* retinas (Figure 8, A and C). Collagen IV accumulation within ECs of *Ephb4^fl/fl^* *Cdh5^ert2cre^* retinas was not readily observed. However, an increased number of collagen IV “empty sleeves” comprising a collagen IV basement membrane without ECs was apparent in *Ephb4^fl/fl^* *Cdh5^ert2cre^* retinas (Figure 8, D and E). This finding is consistent with increased loss of ECs as a result of apoptosis.

### EPHB4 is required for pathological angiogenesis.

**Figure 9:**
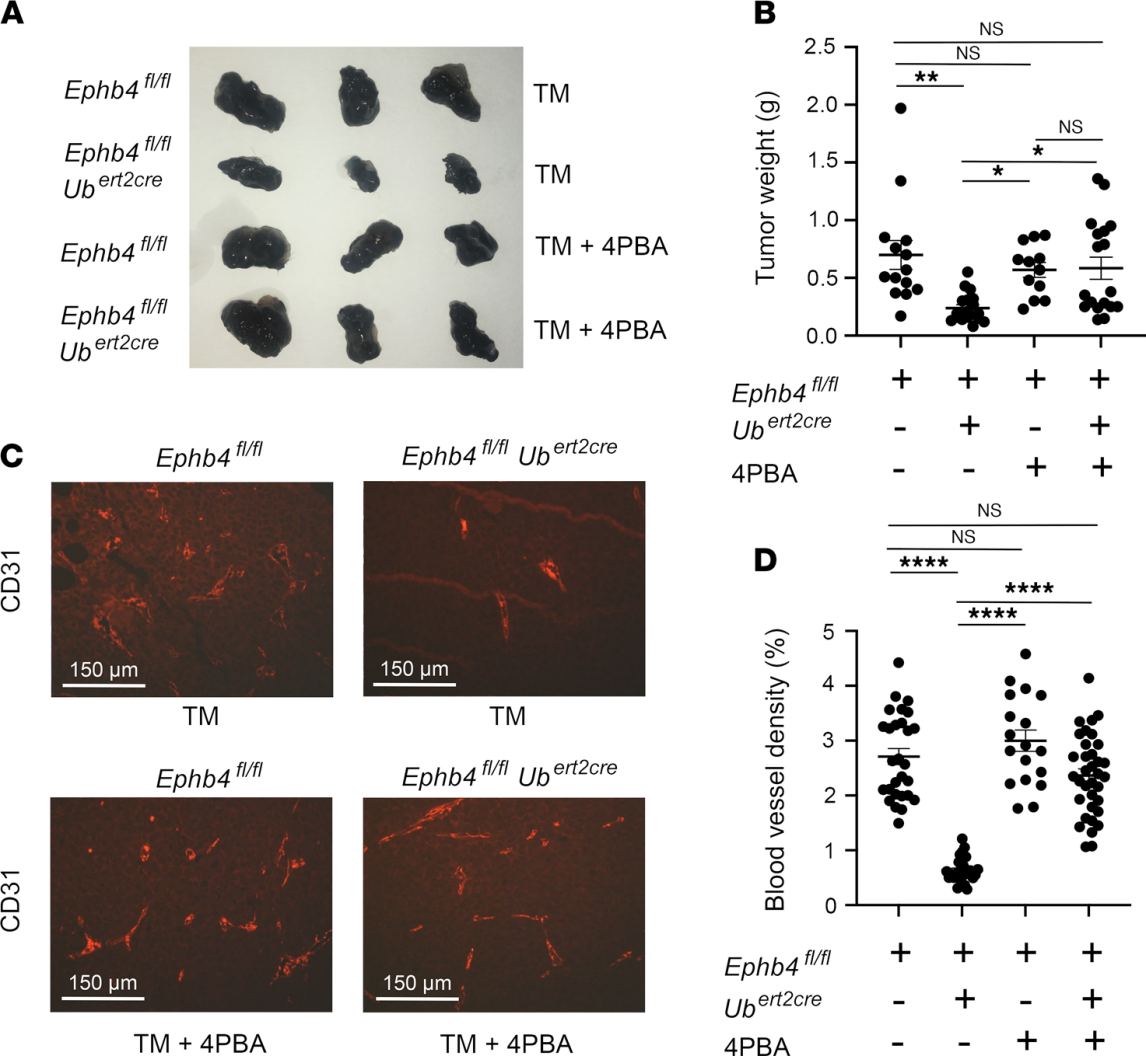
Reduced B16 melanoma growth in adult induced EPHB4-deficient mice associated with impaired tumor angiogenesis. Adult TM-treated *Ephb4^fl/fl^* and *Ephb4^fl/fl^* *Ub^ert2cre^* mice were injected in flanks with B16 melanoma cells and tumors were harvested 13 days later. Some mice received 4PBA at the same time as the tumor and on all subsequent days until tumor harvest. (**A**) Representative images of explanted tumors. (**B**) Plot shows tumor weights at day 13. Bars represent mean ± 1 SEM tumor weight (*Ephb4^fl/fl^* TM alone, *n* = 14; *Ephb4^fl/fl^* *Ub^ert2cre^* TM alone, *n* = 18; *Ephb4^fl/fl^* TM + 4PBA, *n* = 12; *Ephb4^fl/fl^* *Ub^ert2cre^* TM + 4PBA, *n* = 18). (**C**) Sections of explanted tumors were stained with anti-CD31 antibodies to identify BVs. Shown are representative images. (**D**) Plot shows percentage BV coverage of randomly selected 200 × 200 μm areas of tumors. Bars represent mean ± 1 SEM percentage BV coverage (*Ephb4^fl/fl^* TM alone, *n* = 26; *Ephb4^fl/fl^* *Ub^ert2cre^* TM alone, *n* = 27; *Ephb4^fl/fl^* TM + 4PBA, *n* = 18; *Ephb4^fl/fl^* *Ub^ert2cre^* TM + 4PBA, *n* = 35). *, *P* < 0.05; **, *P* < 0.01; ****, *P* < 0.0001; 1-way ANOVA with Tukey.

Pathological angiogenesis in response to tumor growth is another form of neoangiogenesis that is inhibited following induced loss of RASA1 in adult mice (28). We determined if pathological angiogenesis is also impaired in the absence of EPHB4. *Ephb4^fl/fl^* and *Ephb4^fl/fl^* *Ub^ert2cre^* mice were administered TM and were subsequently injected s.c. with B16 melanoma cells. Growth of injected B16 melanoma cells is dependent upon host BV angiogenesis. As determined 13 days later, B16 tumor growth was substantially reduced in *Ephb4^fl/fl^* *Ub^ert2cre^* hosts compared with *Ephb4^fl/fl^* hosts (Figure 9, A and B). Furthermore, reduced tumor growth was associated with a much-reduced BV density in tumor masses explanted from *Ephb4^fl/fl^* *Ub^ert2cre^* hosts (Figure 9, C and D). Notably, administration of 4PBA to *Ephb4^fl/fl^* *Ub^ert2cre^* hosts at the same time as TM and for all subsequent days up to the point of tumor harvest rescued tumor growth, and this was associated with an increased BV density in tumors (Figure 9, A–D). These findings are consistent with the notion that impaired pathological angiogenesis in the absence of EPHB4 is consequent to EC retention of collagen IV.

### Mice that express RASA1 binding–deficient, catalytically active EPHB4 show normal vascular development.

**Figure 10:**
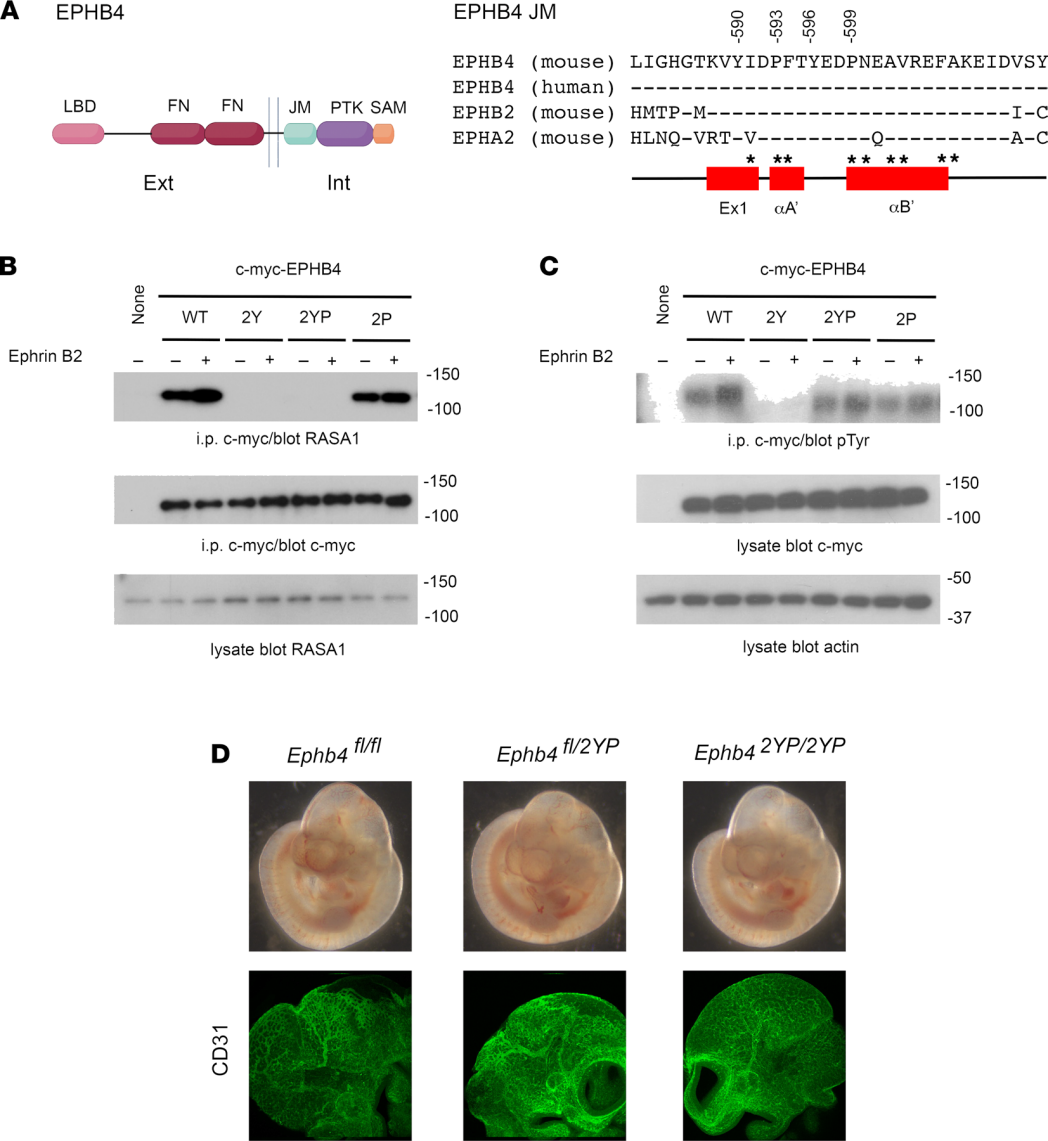
Normal vascular development in homozygous EPHB4 2YP mice. (**A**) At left is a schematic representation of EPHB4 showing the ligand-binding domain (LBD), fibronectin domains (FN), JM segment, PTK domain (PTK), and sterile alpha motif domain (SAM). Ext, extracellular; int, intracellular. At right are shown the amino acid sequences of the JM regions of mouse and human EPHB4 and mouse EPHB2 and EPHA4. Numbering of the indicated conserved tyrosine and proline residues (mutated in EPHB4 2YP) is based upon mouse EPHB4 isoform b. Below are shown the secondary structure elements of the JM segment (27). Ex1, extended strand segment; αA’, single turn helix; αB’, 4 turn helix. Asterisks indicate residues that contact the PTK domain. (**B** and **C**) Cos-7 cells were transfected with c-myc–tagged wild-type (WT), Y590F/Y596F (2Y), Y590F/P593G/Y596F/P599G (2YP), or P593G/P599G (2P) EPHB4. Cells were stimulated or not with Ephrin B2, and transfected EPHB4 receptors were immunoprecipitated from lysates using an anti–c-myc antibody. (**B**) Coimmunoprecipitated RASA1 was detected by Western blotting. (**C**) Phosphotyrosine content of immunoprecipitated EPHB4 was detected by Western blotting. (**D**) Heterozygous *Ephb4^fl/2YP^* mice were intercrossed and embryos were harvested at E10.5. Development of *Ephb4^fl/2YP^* and *Ephb4^2YP/2YP^* was normal at E10.5 (top). Anti-CD31 antibody staining reveals a normal vasculature in the head region of *Ephb4^fl/2YP^* and *Ephb4^2YP/2YP^* embryos (bottom). See complete unedited blots in the supplemental material.

The similarities of vascular phenotype resulting from loss of EPHB4 and RASA1 during embryogenesis prompted us to examine if physical association between RASA1 and EPHB4 is necessary for normal developmental angiogenesis. RASA1 interacts with EPHB4 via SH2 domain-mediated recognition of 2 phosphorylated tyrosine residues, Y590 and Y596, present in the JM region that is highly conserved among Ephrin receptors (Figure 10A) (23, 24). As shown in structural studies of EPHB2 and EPHA4, the JM region normally adopts a helical conformation that interacts with the kinase domain and restrains the kinase in an inactive state (25–27). However, phosphorylation of the analogous JM tyrosine residues in EPHB2 and EPHA4 disrupts the conformation of the JM region, releasing the kinase domain from autoinhibition, resulting in a fully active kinase. With these considerations, simple mutation of Y590 and Y596 to F590 and F596 as a means of specifically disrupting RASA1 interaction with EPHB4 would not represent an informative approach with which to examine a putative role for physical interaction between EPHB4 and RASA1 in vascular development since these mutations would be expected to abrogate EPHB4 kinase activity. To confirm this, wild-type EPHB4 and EPHB4 with Y590F and Y596F mutations were transfected into Cos-7 cells. Cells were then stimulated with Ephrin B2 ligand before immunoprecipitation of transfected EPHB4, followed by Western blotting to detect coimmunoprecipitated RASA1 as well as EPHB4 phosphotyrosine content as a measure of EPHB4 kinase activity. As expected, mutation of both EPHB4 tyrosines resulted in loss of physical interaction between EPHB4 and RASA1. However, the same mutations also resulted in a dead kinase devoid of an ability to mediate EPHB4 tyrosine phosphorylation (Figure 10, B and C). Therefore, to circumvent this problem and generate an EPHB4 receptor that is unable to engage RASA1 but retains kinase activity, we introduced 2 additional mutations, P593G and P599G, into EPHB4 with Y590F and Y596F mutations to generate EPHB4 2YP. In EPHB2 and EPHA4, additional mutation to glycine of the first of these prolines in receptors that contain double tyrosine to phenylalanine mutations restores the kinase activity of these receptors, most likely because of a critical role of this proline residue in stabilizing the helical structure of the JM region necessary for its autoinhibitory activity (27). Similarly, the second of these prolines is predicted to stabilize the helical structure of the JM region (27). In Cos-7 transfection experiments, we confirmed that EPHB4 2YP was unable to bind RASA1 yet retained kinase activity (Figure 10, B and C).

**Figure 11:**
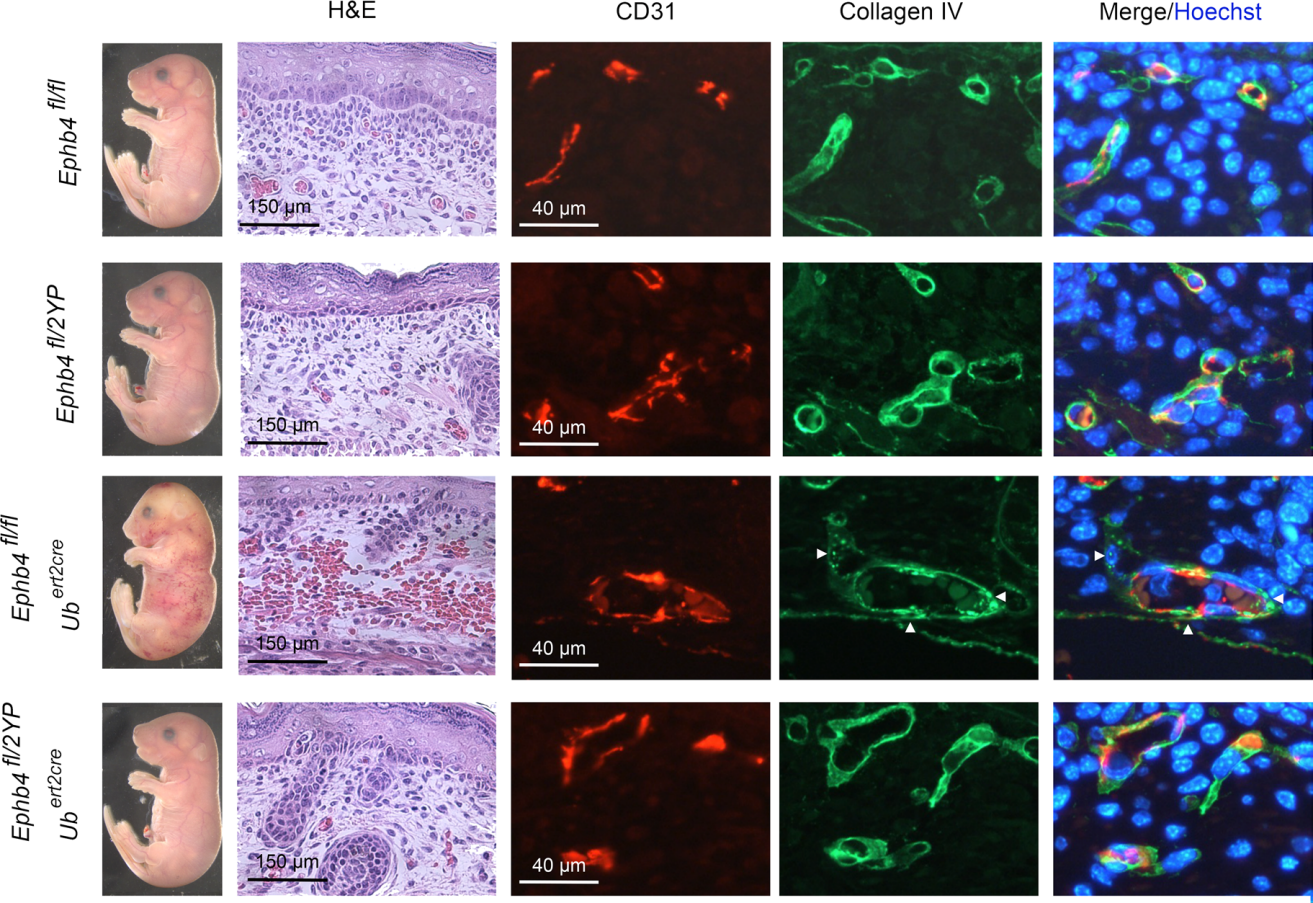
Absence of hemorrhage and EC collagen IV accumulation in ECs of induced EPHB4 2YP embryos. TM was administered to littermate embryos of the indicated genotypes at E13.5 and embryos were harvested at E18.5. Hemorrhage that was confirmed by H&E staining of skin sections was observed in *Ephb4^fl/fl^* *Ub^ert2cre^* embryos but not *Ephb4^fl/2YP^ Ub^ert2cre^* embryos or Cre-negative embryos. Skin sections were additionally stained with anti-CD31 and anti–collagen IV antibodies and Hoechst. Note accumulation of collagen IV in ECs of *Ephb4^fl/fl^* *Ub^ert2cre^* embryos (arrowheads) but not embryos of other genotypes. Scale bars: 150 μm (H&E), 40 μm (anti-CD31).

To examine the impact of EPHB4 2YP upon vascular development, we used CRISPR/Cas9 gene targeting to generate an *Ephb4^2YP^* allele in mice. Unexpectedly, at E10.5, homozygous *Ephb4^2YP/2YP^* mice showed entirely normal vascular development, and disrupted developmental angiogenesis was not evident as it was in constitutive EPHB4-deficient or RASA1-deficient mice (Figure 10D and Table 1). By crossbreeding, we also generated littermate *Ephb4^fl/2YP^* and *Ephb4^fl/fl^* embryos carrying *Ub^ert2Cre^* transgenes. Administration of TM to pregnant dams at E13.5 resulted in EC accumulation of collagen IV and hemorrhage in *Ephb4^fl/fl^* *Ub^ert2Cre^* embryos at E18.5 as noted before (Figure 11 and Table 1). However, neither collagen IV accumulation nor hemorrhage was observed in *Ephb4^fl/2YP^ Ub^ert2Cre^* embryos at E18.5 (Figure 11 and Table 1). These findings show that physical interaction between EPHB4 and RASA1 is not required for normal developmental angiogenesis.

### Physical interaction between EPHB4 and RASA1 is not required for retinal or pathological angiogenesis.

**Figure 12:**
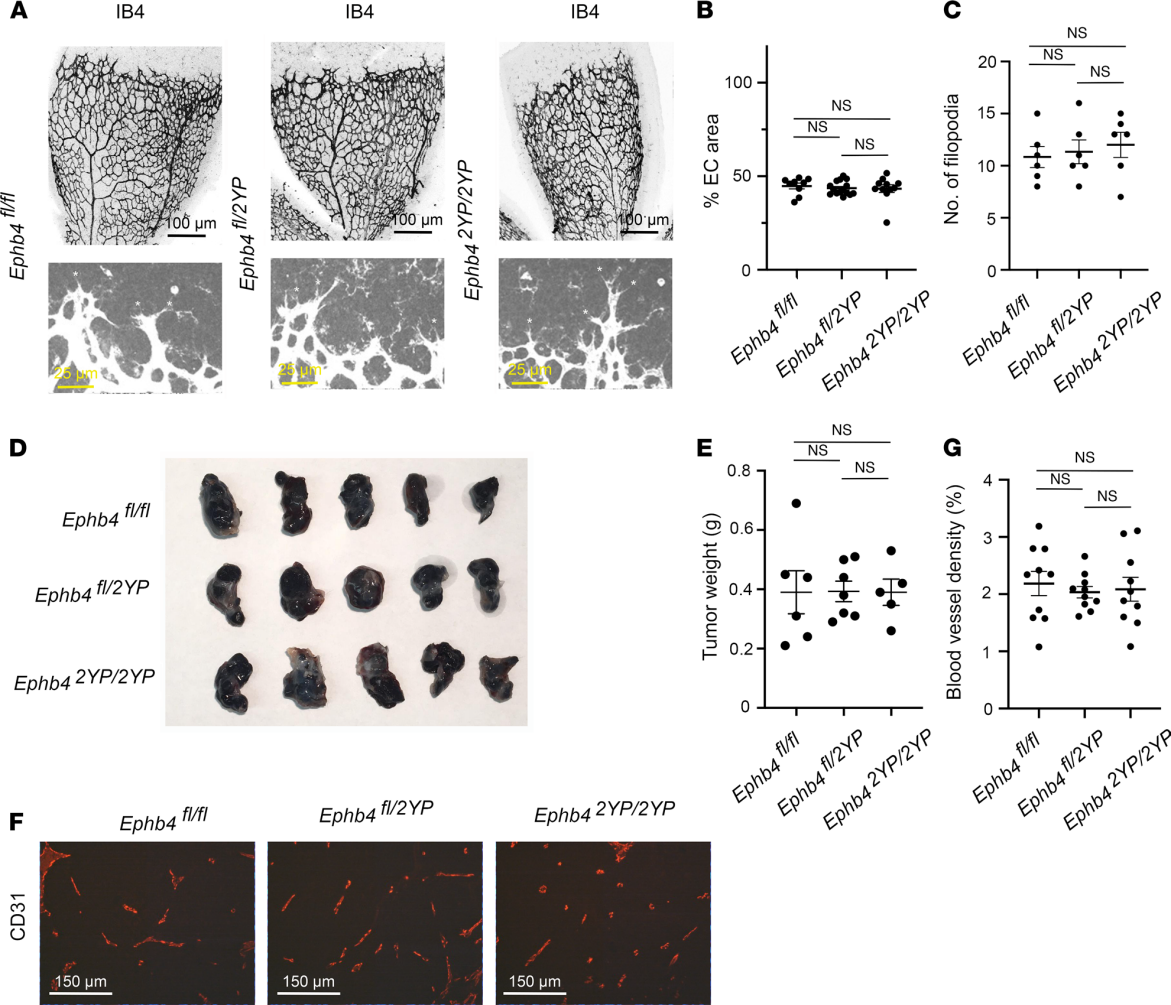
Normal retinal and pathological angiogenesis in EPHB4 2YP mice. (**A**–**C**) Retinas were harvested from mice of the indicated littermate mice at P6 and stained with IB4 to identify BVs. (**A**) Representative low-power images of IB4 staining are shown at top. Representative higher power images of IB4 staining at the angiogenic front are shown at bottom. Asterisks indicate filopodia. Scale bars: 100 μm (top), 25 μm (bottom). (**B**) Plot shows the percentage coverage of retinas with ECs. Bars show the mean ± 1 SEM of percentage EC coverage of individual retinas (*Ephb4^fl/fl^*, *n* = 9; *Ephb4^fl/2YP^*, *n* = 15; *Ephb4^2YP/2YP^*, *n* = 11). (**C**) Plot shows the number of filopodia per 300 μm of angiogenic front that were randomly selected. Bars show the mean ± 1 SEM of filopodia (*Ephb4^fl/fl^*, *n* = 6; *Ephb4^fl/2YP^*, *n* = 6; *Ephb4^2YP/2YP^*, *n* = 6). (**D**–**G**) Adult mice of the indicated genotypes were injected in flanks with B16 melanoma cells, and tumors were harvested 13 days later. (**D**) Representative images of explanted tumors. (**E**) Plot shows tumor weights at day 13. Bars represent mean ± 1 SEM tumor weight (*Ephb4^fl/fl^*, *n* = 6; *Ephb4^fl/2YP^*, *n* = 7; *Ephb4^2YP/2YP^*, *n* = 5). (**E**) Sections of explanted tumors were stained with anti-CD31 antibodies and Hoechst to identify BVs. Shown are representative images. (**F**) Sections of explanted tumors were stained with anti-CD31 antibodies to identify BVs. Shown are representative images. Scale bar: 150 μm. (**G**) Plot shows percentage BV coverage of randomly selected 200 × 200 μm areas of tumors. Bars represent mean ± 1 SEM percentage BV coverage (*Ephb4^fl/fl^*, *n* = 10; *Ephb4^fl/2YP^*, *n* = 10; *Ephb4^2YP/2YP^*, *n* = 10). One-way ANOVA with Tukey.

Consistent with a lack of requirement of physical interaction between EPHB4 and RASA1 for developmental angiogenesis, homozygous *Ephb4^2YP/2YP^* mice survive to adulthood and do not show any spontaneous abnormalities. To examine if physical interaction is required for neonatal angiogenesis, we compared retinal angiogenesis in *Ephb4^fl/fl^*, *Ephb4^fl/2YP^*, and *Ephb4^2YP/2YP^* mice. At P6, no differences in the percentage of EC coverage or the number of filopodia at the angiogenic front were apparent between strains (Figure 12, A–C). We also examined pathological angiogenesis in *Ephb4^fl/fl^*, *Ephb4^fl/2YP^*, and *Ephb4^2YP/2YP^* mice using the B16 melanoma model. Growth of tumors was comparable between the different strains in this model (Figure 12, D and E). In addition, no differences in the density of BVs within tumors were noted between the strains (Figure 12, F and G). We conclude that physical interaction between RASA1 and EPHB4 is not necessary for retinal or pathological angiogenesis.

## Discussion

Accumulating evidence from human studies and animal models indicates that EPHB4 and RASA1 function in the same molecular signaling pathway to regulate BV and LV development and function. In humans, foremost is the finding that inherited inactivating mutations of *EPHB4* and *RASA1* result in CM-AVM (2–4). In addition, inherited inactivating mutations of *EPHB4* are responsible for the development of vein of Galen malformation in humans, a type of brain AVM that has also been described in CM-AVM (2, 3, 36–38). Inherited inactivating mutations in *EPHB4* and *RASA1* are also responsible for the development of lymphatic vascular abnormalities in humans, including lymphedema, chylous ascites and chylothorax, lymphatic-related hydrops fetalis, central conducting lymphatic anomaly, and abnormal lymphatic flow (2, 3, 5–8, 17, 39, 40). In mice, constitutive disruption of *Ephb4* and *Rasa1* results in impaired development of the cardiovascular system and death at E10.5. Furthermore, additional gene-targeting and other studies in mice have revealed a required role for EPHB4 and RASA1 in the development of LV valves, lymphovenous valves, and venous valves and in the maintenance of the adult lymphatic vasculature (16–20, 29, 41).

In this study, we provide further evidence of a functional link between EPHB4 and RASA1 as regulators of the blood vasculature. Specifically, we show that EPHB4, like RASA1, is required for developmental angiogenesis in mid- to late gestation, retinal angiogenesis in newborns, and pathological angiogenesis in adults. Regarding developmental angiogenesis, most importantly, we show that loss of EPHB4 in ECs leads to collagen IV accumulation within the ER and EC apoptotic death. Similarly, loss of RASA1 in ECs during developmental angiogenesis results in accumulation of collagen IV in the ER of ECs and EC apoptotic death (28). In both cases, an inability to export collagen IV for deposition in the nascent basement membrane is mostly likely a consequence of collagen IV misfolding. This is supported by the observation that 4PBA, a small molecular chaperone that facilitates collagen IV folding, promoted collagen IV export from both EPHB4-deficient and RASA1-deficient ECs. Moreover, 4PBA rescued EPHB4-deficient and RASA1-deficient ECs from apoptotic death, thus providing evidence that an inability to export collagen IV is the cause of EC death. Accumulation of collagen IV within the ER could result in EC apoptotic death in 2 distinct ways. First, the misfolded protein could trigger an unfolded protein response that if unable to effectively promote collagen IV folding would lead to apoptosis (31, 42–45). In support of this mechanism, we observed increased abundance of the immunoglobulin binding protein (BiP) ER stress protein in ECs of induced EPHB4-deficient embryos (Supplemental Figure 5). Another possibility is that the paucity of collagen IV in basement membranes following loss of EPHB4 would lead to EC detachment and a default form of apoptosis known as anoikis (46). In our earlier studies of induced RASA1-deficient embryos, both mechanisms were shown to contribute to EC apoptotic death (28).

Previous in vitro studies indicated that in ECs specifically, EPHB4 functions to inhibit Ras/MAPK signaling triggered through other GFRs, such as VEGFR and Tie2 (21, 22). However, whether EPHB4 performs a similar function in ECs in vivo has not been demonstrated before to our knowledge. Therefore, the finding in this study that MAPKs were constitutively active in ECs of induced EPHB4-deficient embryos provides what we believe is the first demonstration of EPHB4’s inhibitory role in Ras/MAPK signaling in ECs in vivo. As in induced RASA1-deficient embryos, an MEK inhibitor was able to partially rescue developmental vascular phenotypes in induced EPHB4-deficient embryos. This finding is consistent with a central role for dysregulated Ras/MAPK signaling in EC collagen IV accumulation and downstream sequelae. In the absence of RASA1, dysregulated Ras/MAPK signaling is associated with increased expression of proline and lysine hydroxylases in the ER that are predicted to cause excessive hydroxylation of collagen IV that could account for collagen IV misfolding (28). We propose that a similar Ras/MAPK-driven increased abundance of collagen IV–modifying proline and lysine hydroxylases is responsible for collagen IV misfolding and accumulation in the absence of EPHB4. Hence, 2,4PDCA, a broad inhibitor of the family of 2OG-dependent oxygenases to which collagen IV proline and lysine hydroxylases belong, promotes collagen IV export in induced EPHB4-deficient embryos as it does in induced RASA1-deficient embryos.

Further suggestive of a functional relationship between EPHB4 and RASA1 is the known physical association between RASA1 and EPHB4 mediated by RASA1 SH2 domain recognition of 2 phosphorylated tyrosine residues located in the JM segment of EPHB4 (22–24). To address the significance of this interaction for vascular development and function, we generated knockin mice that expressed a 2YP mutant of EPHB4 that is unable to bind RASA1 but retains PTK activity. Surprisingly, homozygous EPHB4 2YP mice showed entirely normal developmental, neonatal, and pathological angiogenesis. Thus, a model in which EPHB4 functions as a docking receptor to recruit RASA1 to the plasma membrane whereupon it might become juxtaposed to Ras, permitting Ras inactivation, is probably incorrect. How EPHB4 and RASA1 cooperate to negatively regulate Ras in ECs remains to be determined. In CM-AVM1, missense RASA1 mutations have been reported that are located mostly within the pleckstrin homology and C2 homology domains of RASA1 (2, 3). These domains coordinate binding to phospholipids, suggesting that membrane targeting of RASA1 may be mediated primarily through lipid recognition rather than receptor interaction (47). EPHB4 may be uniquely involved in the generation of phospholipid ligands of RASA1.

A requirement for EPHB4 and RASA1 for BV angiogenesis but not maintenance of BVs in adults is mostly likely explained by the stability of collagens (35). In this regard, sufficient collagen IV may be deposited in basement membranes during developmental angiogenesis that obviates a requirement thereafter for blood ECs to continue to engage in high-rate synthesis of collagen IV to remain attached to vessel walls. Accordingly, loss of EPHB4 or RASA1 would not affect BVs past the stage of developmental angiogenesis, except where de novo synthesis of collagen IV is required, e.g., retinal angiogenesis and pathological angiogenesis. The finding that RASA1 and EPHB4 are required for the maintenance of venous valves and LV valves in adults is also consistent with this hypothesis (16, 20, 28). Valvular ECs would be subject to higher shear stress forces than lumenal wall ECs that might necessitate their continued high-rate synthesis of collagen IV for them to remain attached to valve leaflets (48, 49).

The findings reported herein for EPHB4 and reported for RASA1 previously are relevant to an understanding of the pathogenesis of fast flow lesions in CM-AVM. One possibility is that somatic second hit mutation of *EPHB4* or *RASA1* in patients with germline *EPHB4* or *RASA1* mutations, respectively, occurs in isolated ECs during vasculogenesis such that the majority of ECs within a single vessel within a primitive vascular plexus are EPHB4 or RASA1 null. As such, remodeling of that vessel during developmental angiogenesis would not be possible since this would be dependent upon an ability of ECs within that vessel to deposit collagen IV in a nascent basement membrane. The result would be a direct connection between arteries and veins without an intervening capillary bed that constitutes the quintessential feature of an AVM. However, another possibility is that EPHB4- or RASA1-null ECs generated by a second hit mutation during vasculogenesis or developmental angiogenesis are rescued from apoptosis by neighboring ECs that have not acquired second hit mutations and continue to express EPHB4 or RASA1. These neighboring ECs could provide sufficient local collagen IV in basement membranes to rescue adjacent EPHB4- or RASA1-deficient ECs from anoikis. In this model, dysregulated Ras/MAPK signaling in EPHB4- or RASA1-null ECs could drive AVM formation through a distinct mechanism. Which of these models is correct will have bearing upon the ability of drugs that inhibit Ras/MAPK signaling or promote collagen IV folding to prevent the development of and to treat vascular lesions in this disease.

## Methods

### Mice.

Mice carrying *exon 2–exon 3* floxed alleles of *Ephb4* and *exon 1* floxed alleles of *Ephb4* have been described (17, 50). Mice were crossed with *Ub^ert2cre^* and *Cdh5^ert2cre^* transgenic mice to generate littermate *Ephb4^fl/fl^* progeny with and without either *cre* transgene. Mice were on mixed 129S6/SvEv C57BL/6 genetic backgrounds originally from The Jackson Laboratory.

Mice carrying a knockin *Ephb4 Y590F/P593G/Y596F/P599G* (*Ephb4 2YP*) allele (numbering based on transcript variant 2, NM_010144.6; isoform b, NP_034274.4) were generated using CRISPR/Cas9 technology. Exon 11 of Ensembl gene model Transcript Ephb4-203 (ENSMUST00000111055.9) includes codons Y599, P602, U605, and P608. The CRISPOR algorithm (51) was used to identify specific single-guide RNAs (sgRNAs). sgRNAs predicted to cut the chromosome near codon 608 were tested to determine if they caused chromosome breaks. sgRNAs were chemically synthesized with phosphorothioate modifications by MilliporeSigma (52, 53). Enhanced specificity *Streptomyces pyogenes* Cas9 endonuclease protein (ESPCAS9) (54) was obtained from MilliporeSigma. sgRNAs (30 ng/μL) were complexed with ESPCAS9 (50 ng/μL) and individually tested to determine if the ribonucleoprotein (RNP) complexes caused chromosome breaks in mouse zygotes. RNPs were microinjected into fertilized mouse eggs. Eggs were placed in culture until they developed into blastocysts. DNA was extracted from individual blastocysts for analysis. PCR with primers spanning the predicted cut site was used to generate amplicons for Sanger sequencing. The process was essentially as described (55). sgRNA candidates were tested with forward primer 5′ GTATGACTCAGTTTGCCTTTTGCTTCTTT 3′ and reverse primer 5*′* TTTTCAGTAATTAGTTCTCTCCTCCCAGC 3*′*, 788 bp amplicon. Sequence chromatograms of amplicons from individual blastocysts were evaluated to determine if small insertions/deletions caused by nonhomologous end joining repair of chromosome breaks were present. sgRNA C248G2 was found to induce chromosome breaks. It targets the sequence 5′ TACTTACGAAGACCCTAATG (protospace adjacent motif = AGG) 3′ (cutting frequency determination score of 86) (56).

After determining that C248G2 caused chromosome breaks, we combined RNPs with 10 ng/μL single-stranded oligonucleotide DNA donor (IDT, https://www.idtdna.com/pages). The sequence of the single-stranded oligonucleotide DNA donor was CAGAGGAATTTACTTCCTGGTTAATGGGCTCCTGTGTGACTCCTTAGGTACCAAGGTCTtCATTGATggcTTTACTTtCGAAGACggcAATGAGGCAGTGAGGGAATTTGCCAAAGAGATCGATGTCTCCTATGTCAAGATTGAAGAG (lowercase letters indicate coding changes to the wild-type exon; exon 11 is underlined). The CRISPR reagents were microinjected into fertilized mouse eggs produced by mating superovulated B6SJLF1 female mice (The Jackson Laboratory stock no. 100012) to B6SJLF1 male mice as described (57). CRISPR/Cas9 microinjection of 325 B6SJLF1 zygotes produced 79 potential founder mice. Five generation 0 founder (G0) pups were identified by Sanger sequencing of amplicons spanning exon 11. G0 founders were mated with wild-type C57BL/6J mice (The Jackson Laboratory) to obtain germline transmission of the *Eph4b* mutant. Following germline transmission, we confirmed correct targeting and an absence of incorrect targeting at several other possible loci identified by CRISPOR (*Epha2*, *Epha4*, *Ephb1*, *Ephb2*, *Nfat5*, *PYKFyve2*, and *Plxna2*) by Sanger sequencing (58). Heterozygous *Ephb4^2YPG^* mice were crossed with *Ephb4^fl^ exon 2–3* mice and *Ub^ert2cre^* mice for studies.

### Developmental angiogenesis.

To induce embryonic disruption of *Ephb4*, pregnant mice carrying embryos with *Ephb4^fl^* alleles were given 3 i.p. injections of TM (MilliporeSigma; 0.05 mg/g body weight per injection, dissolved in corn oil) on consecutive days. The MEK inhibitor AZD6244 (Selleckchem; 0.05 mg/g body weight per injection), the chemical chaperone 4PBA (MilliporeSigma; 0.25 mg/g body weight per injection), and the 2OG-dependent oxygenase inhibitor 2,4PDCA (MilliporeSigma; 0.1 mg/g body weight per injection) were injected i.p. into mice at the same time as TM and on all subsequent days of gestation until embryo harvest. Embryos were fixed in 3.75% formaldehyde overnight and embedded in paraffin. Five-micrometer sections were dehydrated and antigen retrieval was performed with a Diva decloaking kit (Biocare Medical). Sections were blocked in PBS/10% donkey serum/0.3% Triton X-100 and incubated overnight with the following primary antibodies in PBS in 10% donkey serum: rat anti-CD31 (clone SZ31, Dianova), rabbit anti–active caspase 3 (catalog AF835, R&D Systems, Bio-Techne), goat anti–collagen IV (catalog 1340-01, SouthernBiotech), rabbit anti-BiP (catalog 3177, Cell Signaling Technology), rabbit anti-TGN46 (catalog ab16059, Abcam), rabbit anti-LMNA1 (catalog ab125006, Abcam), rabbit anti-calnexin (catalog ab22595, Abcam), rabbit anti–LYVE-1 (catalog ab14917, Abcam), rabbit anti-calreticulin (clone D3E6, Cell Signaling Technology), rat anti-LAMP1 (clone 1D4B, Thermo Fisher Scientific), and rabbit anti-phospho-ERK (clone D13.14.4E, Cell Signaling Technology). Secondary antibodies used were species-specific anti-immunoglobulin donkey F(ab′)_2_ fragments coupled to Alexa Fluor 488, 594, or 647 (Jackson ImmunoResearch, catalog 705-546-147, 711-546-152, 712-586-150, 705-586-147, 712-606-153, 711-606-152, 715-586-152; Invitrogen, Thermo Fisher Scientific, catalog A-21208; SouthernBiotech, catalog 6430-31) and were incubated with tissues in PBS for 2 hours. Sections were stained with Hoechst (Invitrogen, Thermo Fisher Scientific) to identify nuclei before mounting and viewing on a Leica SP5 X confocal microscope (Leica Microsystems).

For whole mount staining of E10.5 embryos from *Ephb4^fl/2YP^* intercrosses, embryos were fixed in 1% paraformaldehyde for 1 hour, blocked in 3% donkey serum/0.3% Triton X-100 in PBS overnight, and stained with anti-CD31 antibody (clone MEC 13.3, eBioscience, Thermo Fisher Scientific) in 0.3% Triton X-100 in PBS overnight. Embryos were then incubated with Alexa Fluor 488 donkey anti–rat Ig overnight before mounting and viewing on a BX60 upright fluorescence microscope (Nikon).

### Retinal angiogenesis.

Newborn pups were injected with TM (0.05 mg/g body weight per injection) on 2 consecutive days from P1–P2 and retinas were harvested at P6. Retinas from pups of *Ephb4^fl/2YP^* intercrosses were also harvested at P6. Retinas were fixed in 4% paraformaldehyde for 2 hours, then were blocked in PBS/10% donkey serum and incubated overnight with IB4-FITC (catalog 217660, MilliporeSigma) and goat anti–collagen IV in PBS/10% donkey serum. Retinas were subsequently incubated for 2 hours with a secondary anti-goat donkey F(ab′)_2_ coupled to Alexa Fluor 594 in PBS to detect collagen IV. Whole mounts were viewed on a BX60 upright fluorescence microscope. The percentage EC coverage and number of empty sleeves per field were determined in randomly chosen 50 × 50 μm areas behind the angiogenic front in a region between an artery and a vein. The number of filopodia at the angiogenic front was determined for randomly chosen 300 μm regions.

### Tumor angiogenesis.

TM was administered to male and female mice at 2 months of age. After 2 weeks, mice were injected s.c. in the flank with 0.75 × 10^6^ B16F10 cells (provided by Weiping Zou, Michigan Medicine, Ann Arbor, Michigan, USA) suspended in 100 μL matrigel (Corning). Chemical chaperone 4PBA was administered to some mice at the same time as tumor cells and for each day until tumor harvest at day 13 (0.4 mg/g body weight per injection). Tumors were fixed in 4% paraformaldehyde overnight, and 5 μm sections were stained with Hoechst and antibodies against CD31. Sections were viewed on a BX60 upright fluorescence microscope or a Leica SP5 X confocal microscope. BV density was assessed within random 200 × 200 μm regions of tumor sections.

### EPHB4 physical association with RASA1 and EPHB4 kinase activity.

C-myc–tagged murine *Ephb4* cDNA in pCMV6 was purchased from Origene. Double Y590F/Y596F (2Y) and P593G/P599G (2P) and quadruple Y590F/P593G/Y596F/P599G (2YP) EPHB4 mutations were introduced by site-directed mutagenesis using a QuikChange Lightning Multi Site-Directed Mutagenesis Kit (Agilent) according to the manufacturer’s instructions. Cos-7 cells (ATCC) at 80% confluence in 10 cm culture dishes were transfected with 10 μg of plasmids using Lipofectamine in Opti-MEM (both from Thermo Fisher Scientific). After 48 hours, cells were serum-starved in DMEM for 16 hours before stimulation with Ephrin B2 Fc chimeric protein (1 μg/mL; R&D Systems, Bio-Techne) for 15 minutes. Cells were lysed in RIPA buffer (50 mM Tris-HCl at pH 7.4, 150 mM NaCl, 1% NP-40, 0.5% Na-deoxycholate, 1 mM EDTA), and lysates were incubated with anti–c-myc antibody (clone 9E10; MilliporeSigma) overnight, followed by rotation with Protein A/G PLUS-Agarose beads (Santa Cruz Biotechnology) for 2 hours. After washing, beads were boiled in 1× SDS sample buffer, and eluates were run on 10% SDS-PAGE gels. Coimmunoprecipitated RASA1 was detected by Western blotting using an anti-RASA1 antibody (clone B4F8; Santa Cruz Biotechnology) and secondary goat anti–mouse IgG linked to horseradish peroxidase (GAM-HRP; catalog 91196, Cell Signaling Technology). Phosphotyrosine content of immunoprecipitated EPHB4 was determined by Western blotting using an anti-phosphotyrosine antibody (clone 4G10; MilliporeSigma) and GAM-HRP. Equivalent transfection of Cos-7 cells was shown by Western blotting of immunoprecipitates and whole-cell lysates using 9E10 and GAM-HRP.

### Western blot analysis of MAPK activation.

Fetal liver was harvested from E18.5 embryos, and whole-cell lysates were prepared in RIPA buffer. MAPK activation was determined by Western blotting using an anti–phospho-ERK antibody (clone D13.14.E, Cell Signaling Technology) and GAM-HRP. Total ERK content was determined by Western blotting using an anti-ERK antibody (clone 137F5, Cell Signaling Technology) and goat anti–rabbit IgG–HRP (catalog 7074, Cell Signaling Technology).

### Statistics.

For quantitative assessment of embryonic vascular development, replicate determinations were obtained from at least 2 embryos for each genotype and condition. For quantitative assessment of retina angiogenesis, replicate determinations were obtained from all examined retinas (Figure 8) or 6 examined retinas of each genotype (Figure 12). For quantitative assessment of tumor angiogenesis, replicate determinations were obtained from all examined tumors (Figures 8 and 12). Statistical analysis was performed using Mann-Whitney nonparametric tests, Student’s 2-sample 2-tailed *t* tests, and 1-way ANOVA tests with Tukey’s correction as indicated. A *P* value less than 0.05 was considered significant.

### Study approval.

All experiments performed with mice were in compliance with University of Michigan guidelines and were approved by the university IACUC.

## Author contributions

DC and PDK contributed to the design of studies. JW, MNHV, and TM provided Ephb4 conditional mouse models. EDH and TLS assisted with the generation of Ephb4-knockin mice. DC performed experiments. The manuscript was written by DC and PDK.

## Supplementary Material

Supplemental data

## Figures and Tables

**Table 1:**
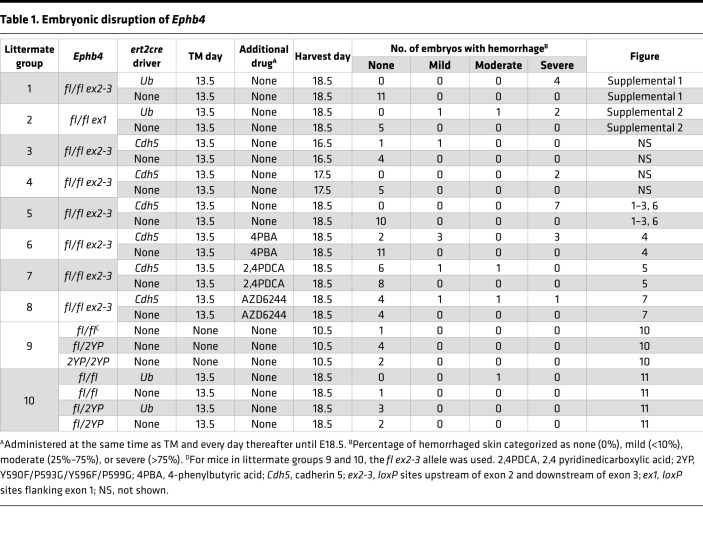
Embryonic disruption of *Ephb4*

## Version 1 01/11/2022

In-Press Preview

## Version 2 02/22/2022

Electronic publication

## References

[1] Eerola I (2003). Capillary malformation-arteriovenous malformation, a new clinical and genetic disorder caused by RASA1 mutations. Am J Hum Genet.

[2] Revencu N (2013). RASA1 mutations and associated phenotypes in 68 families with capillary malformation-arteriovenous malformation. Hum Mutat.

[3] Revencu N (2008). Parkes Weber syndrome, vein of Galen aneurysmal malformation, and other fast-flow vascular anomalies are caused by RASA1 mutations. Hum Mutat.

[4] Amyere M (2017). Germline loss-of-function mutations in EPHB4 cause a second form of capillary malformation-arteriovenous malformation (CM-AVM2) deregulating RAS-MAPK signaling. Circulation.

[5] Burrows PE (2013). Lymphatic abnormalities are associated with RASA1 gene mutations in mouse and man. Proc Natl Acad Sci U S A.

[6] de Wijn RS (2012). Phenotypic variability in a family with capillary malformations caused by a mutation in the RASA1 gene. Eur J Med Genet.

[7] Macmurdo CF (2016). RASA1 somatic mutation and variable expressivity in capillary malformation/arteriovenous malformation (CM/AVM) syndrome. Am J Med Genet A.

[8] Sevick-Muraca EM, King PD (2014). Lymphatic vessel abnormalities arising from disorders of Ras signal transduction. Trends Cardiovasc Med.

[9] Gasper R, Wittinghofer F (2019). The Ras switch in structural and historical perspective. Biol Chem.

[10] King PD (2013). Nonredundant functions for Ras GTPase-activating proteins in tissue homeostasis. Sci Signal.

[11] Lapinski PE (2018). Somatic second hit mutation of RASA1 in vascular endothelial cells in capillary malformation-arteriovenous malformation. Eur J Med Genet.

[12] Revencu N (2020). RASA1 mosaic mutations in patients with capillary malformation-arteriovenous malformation. J Med Genet.

[13] Adams RH (1999). Roles of ephrinB ligands and EphB receptors in cardiovascular development: demarcation of arterial/venous domains, vascular morphogenesis, and sprouting angiogenesis. Genes Dev.

[14] Gerety SS (1999). Symmetrical mutant phenotypes of the receptor EphB4 and its specific transmembrane ligand ephrin-B2 in cardiovascular development. Mol Cell.

[15] Henkemeyer M (1995). Vascular system defects and neuronal apoptosis in mice lacking ras GTPase-activating protein. Nature.

[16] Lapinski PE (2017). RASA1 regulates the function of lymphatic vessel valves in mice. J Clin Invest.

[17] Martin-Almedina S (2016). EPHB4 kinase-inactivating mutations cause autosomal dominant lymphatic-related hydrops fetalis. J Clin Invest.

[18] Chen D (2020). RASA1-driven cellular export of collagen IV is required for the development of lymphovenous and venous valves in mice. Development.

[19] Frye M (2020). EphrinB2-EphB4 signalling provides Rho-mediated homeostatic control of lymphatic endothelial cell junction integrity. Elife.

[20] Lyons O (2021). Mutations in EPHB4 cause human venous valve aplasia. JCI Insight.

[21] Xiao Z (2012). EphB4 promotes or suppresses Ras/MEK/ERK pathway in a context-dependent manner: implications for EphB4 as a cancer target. Cancer Biol Ther.

[22] Kim I (2002). EphB ligand, ephrinB2, suppresses the VEGF- and angiopoietin 1-induced Ras/mitogen-activated protein kinase pathway in venous endothelial cells. FASEB J.

[23] Holland SJ (1997). Juxtamembrane tyrosine residues couple the Eph family receptor EphB2/Nuk to specific SH2 domain proteins in neuronal cells. EMBO J.

[24] Kawasaki J (2014). RASA1 functions in EPHB4 signaling pathway to suppress endothelial mTORC1 activity. J Clin Invest.

[25] Binns KL (2000). Phosphorylation of tyrosine residues in the kinase domain and juxtamembrane region regulates the biological and catalytic activities of Eph receptors. Mol Cell Biol.

[26] Wiesner S (2006). A change in conformational dynamics underlies the activation of Eph receptor tyrosine kinases. EMBO J.

[27] Wybenga-Groot LE (2001). Structural basis for autoinhibition of the Ephb2 receptor tyrosine kinase by the unphosphorylated juxtamembrane region. Cell.

[28] Chen D (2019). RASA1-dependent cellular export of collagen IV controls blood and lymphatic vascular development. J Clin Invest.

[29] Lapinski PE (2012). RASA1 maintains the lymphatic vasculature in a quiescent functional state in mice. J Clin Invest.

[30] Udan RS (2013). Understanding vascular development. Wiley Interdiscip Rev Dev Biol.

[31] Jeanne M (2015). Molecular and genetic analyses of collagen type IV mutant mouse models of spontaneous intracerebral hemorrhage identify mechanisms for stroke prevention. Circulation.

[32] Kuo DS (2014). Allelic heterogeneity contributes to variability in ocular dysgenesis, myopathy and brain malformations caused by Col4a1 and Col4a2 mutations. Hum Mol Genet.

[33] Rose NR (2011). Inhibition of 2-oxoglutarate dependent oxygenases. Chem Soc Rev.

[34] Luxan G (2019). Endothelial EphB4 maintains vascular integrity and transport function in adult heart. Elife.

[35] Shoulders MD, Raines RT (2009). Collagen structure and stability. Annu Rev Biochem.

[36] Duran D (2019). Mutations in chromatin modifier and ephrin signaling genes in vein of Galen malformation. Neuron.

[37] Vivanti A (2018). Loss of function mutations in EPHB4 are responsible for vein of Galen aneurysmal malformation. Brain.

[38] Zeng X (2019). EphrinB2-EphB4-RASA1 signaling in human cerebrovascular development and disease. Trends Mol Med.

[39] Martin-Almedina S (2021). Janus-faced EPHB4-associated disorders: novel pathogenic variants and unreported intrafamilial overlapping phenotypes. Genet Med.

[40] Li D (2018). Pathogenic variant in EPHB4 results in central conducting lymphatic anomaly. Hum Mol Genet.

[41] Zhang G (2015). EphB4 forward signalling regulates lymphatic valve development. Nat Commun.

[42] Guiraud S (2017). HANAC Col4a1 mutation in mice leads to skeletal muscle alterations due to a primary vascular defect. Am J Pathol.

[43] Jeanne M (2012). COL4A2 mutations impair COL4A1 and COL4A2 secretion and cause hemorrhagic stroke. Am J Hum Genet.

[44] Kim I (2008). Cell death and endoplasmic reticulum stress: disease relevance and therapeutic opportunities. Nat Rev Drug Discov.

[45] Oslowski CM, Urano F (2010). The binary switch between life and death of endoplasmic reticulum-stressed beta cells. Curr Opin Endocrinol Diabetes Obes.

[46] Michel JB (2003). Anoikis in the cardiovascular system: known and unknown extracellular mediators. Arterioscler Thromb Vasc Biol.

[47] Lemmon MA (2008). Membrane recognition by phospholipid-binding domains. Nat Rev Mol Cell Biol.

[48] Bazigou E, Makinen T (2013). Flow control in our vessels: vascular valves make sure there is no way back. Cell Mol Life Sci.

[49] Bazigou E (2014). Primary and secondary lymphatic valve development: molecular, functional and mechanical insights. Microvasc Res.

[50] Wang Y (2015). EPHB4 protein expression in vascular smooth muscle cells regulates their contractility, and EPHB4 deletion leads to hypotension in mice. J Biol Chem.

[51] Haeussler M (2016). Evaluation of off-target and on-target scoring algorithms and integration into the guide RNA selection tool CRISPOR. Genome Biol.

[52] Basila M (2017). Minimal 2’-O-methyl phosphorothioate linkage modification pattern of synthetic guide RNAs for increased stability and efficient CRISPR-Cas9 gene editing avoiding cellular toxicity. PLoS One.

[53] Hendel A (2015). Chemically modified guide RNAs enhance CRISPR-Cas genome editing in human primary cells. Nat Biotechnol.

[54] Slaymaker IM (2016). Rationally engineered Cas9 nucleases with improved specificity. Science.

[55] Sakurai T (2014). A single blastocyst assay optimized for detecting CRISPR/Cas9 system-induced indel mutations in mice. BMC Biotechnol.

[56] Doench JG (2016). Optimized sgRNA design to maximize activity and minimize off-target effects of CRISPR-Cas9. Nat Biotechnol.

[57] Van Keuren ML (2009). Generating transgenic mice from bacterial artificial chromosomes: transgenesis efficiency, integration and expression outcomes. Transgenic Res.

[58] Concordet JP, Haeussler M (2018). CRISPOR: intuitive guide selection for CRISPR/Cas9 genome editing experiments and screens. Nucleic Acids Res.

