# Supplementary material for: Angiogenesis depends upon EPHB4-mediated export of collagen IV from vascular endothelial cells
Source: JCI Insight. 2022 Feb 22;7(4):e156928. doi: 10.1172/jci.insight.156928 (PMC8876457; doi:10.1172/jci.insight.156928)

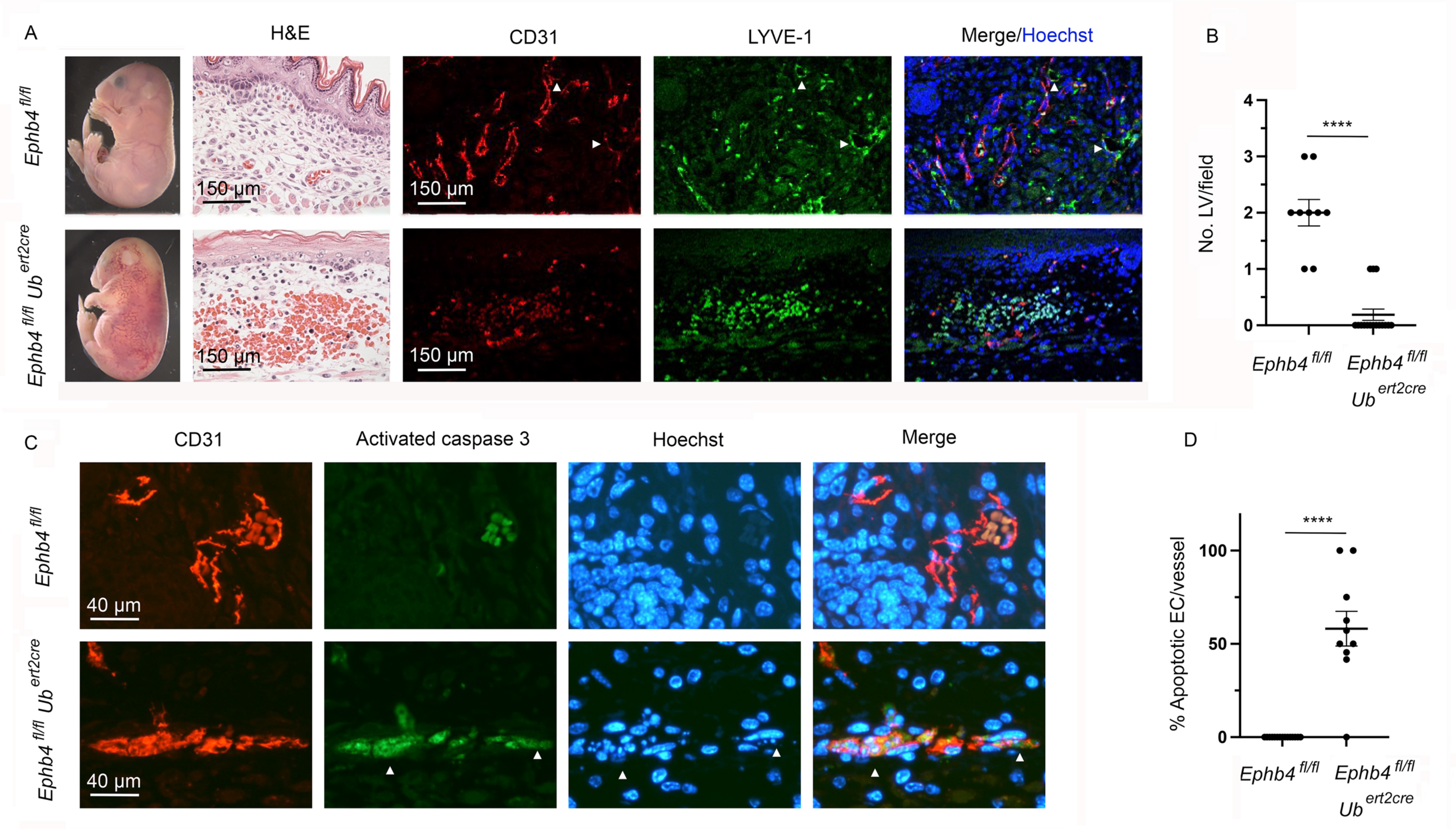

Supplemental Figure 1. Hemorrhage, EC apoptosis and reduced lymphatic vessels following induced global disruption of *Ephb4* during developmental angiogenesis. TM was administered to *Ephb4*$^{fl/fl}$ and *Ephb4*$^{fl/fl}$ *Ub*^{ert2cre} embryos at E13.5 and embryos were harvested at E18.5. (A) Images at left show extensive cutaneous hemorrhage and an edematous appearance of *Ephb4*$^{fl/fl}$ *Ub*^{ert2cre} embryos. H&E staining of skin sections confirmed vascular hemorrhage and combined CD31 and LYVE-1 staining of sections revealed a reduced number of intact CD31^{lo}LYVE-1+ initial LV in *Ephb4*$^{fl/fl}$ *Ub*^{ert2cre} embryos (shown with arrowheads in images of *Ephb4*$^{fl/fl}$ sections). (B) Plot shows the number of identified CD31^{lo}LYVE-1+ LV in randomly selected 200 µm x 200 µm areas of skin of *Ephb4*$^{fl/fl}$ and *Ephb4*$^{fl/fl}$ *Ub*^{ert2cre} embryos. Bars show the mean +/- 1 SEM of LV/field (*Ephb4*$^{fl/fl}$, n=9; *Ephb4*$^{fl/fl}$ *Ub*^{ert2cre}, n=16). (C) Skin sections were stained with anti-CD31 and anti-activated caspase 3 antibodies and Hoecsht to identify apoptotic EC. Examples of activated caspase 3-positive EC with fragmented nuclei in images of *Ephb4*$^{fl/fl}$ *Ub*^{ert2cre} sections are indicated with arrowheads. (D) Plot shows the percentage of apoptotic cells in individual CD31+ BV in skin of *Ephb4*$^{fl/fl}$ and *Ephb4*$^{fl/fl}$ *Ub*^{ert2cre} embryos selected from multiple randomly chosen areas. Bars show the mean +/- 1 SEM of percentage apoptotic EC per vessel (*Ephb4*$^{fl/fl}$, n=15; *Ephb4*$^{fl/fl}$ *Ub*^{ert2cre}, n=10). ****, $P<0.0001$; Mann Whittney test.

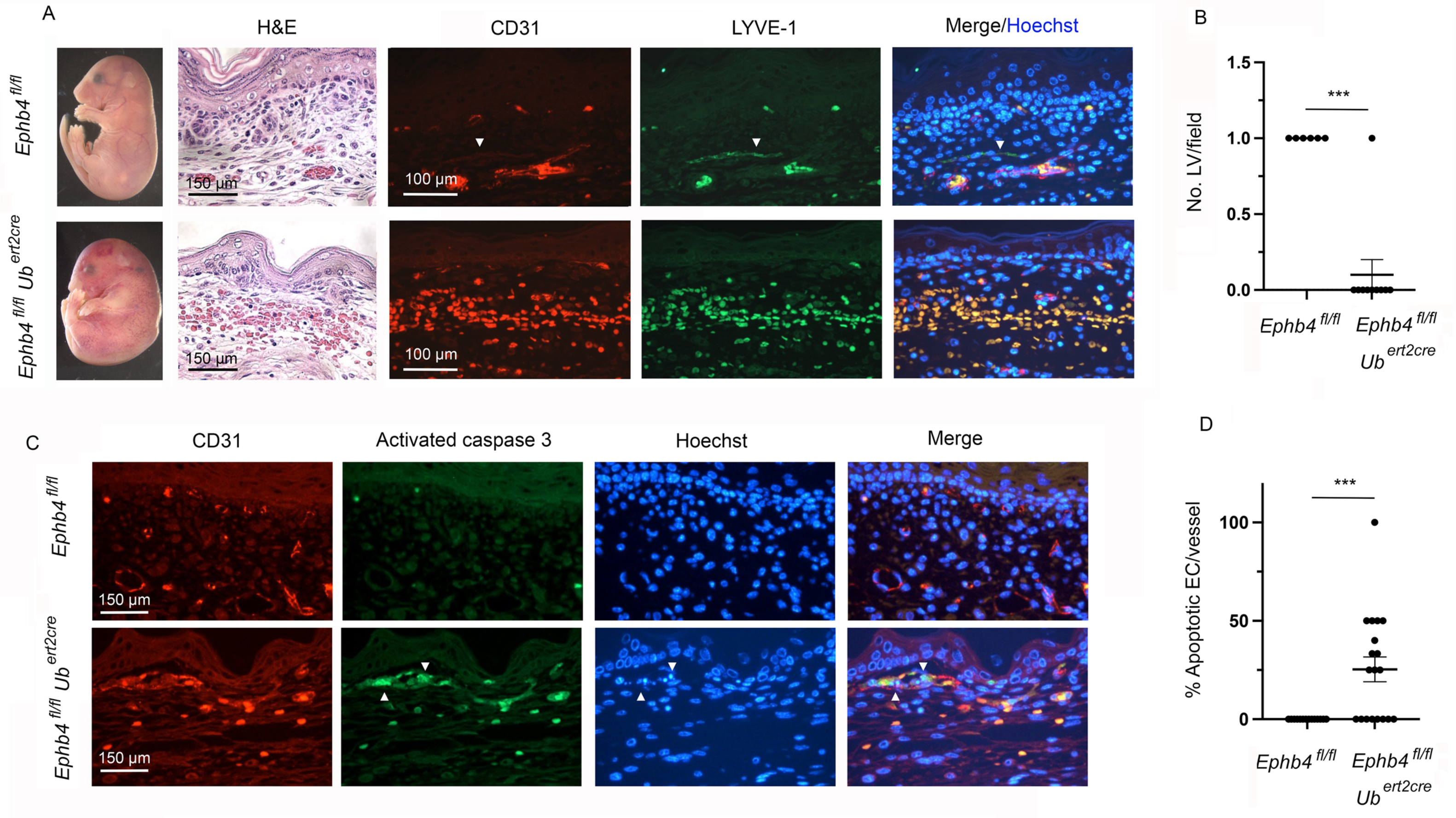

Supplemental Figure 2. Disruption of an exon 1 floxed *Ephb4* allele during developmental angiogenesis results in EC apoptosis, hemorrhage and reduced LV density. *Ephb4$^{fl/fl}$* and *Ephb4$^{fl/fl}$ Ub^{ert2cre}* embryos in which exon 1 of *Ephb4* was floxed were administered TM at E13.5 and harvested at E18.5. (A) Extensive skin hemorrhage in *Ephb4$^{fl/fl}$ Ub^{ert2cre}* embryos confirmed by H&E staining and reduced intact CD31^{lo}LYVE-1+ initial LV shown by staining with anti-CD31 and anti-LYVE-1 antibodies (shown with arrowheads in images of *Ephb4$^{fl/fl}$* sections). (B) Plot shows the number of identified CD31^{lo} LYVE-1+ LV in randomly selected 200 µm x 200 µm areas of skin of *Ephb4$^{fl/fl}$* and *Ephb4$^{fl/fl}$ Ub^{ert2cre}* embryos. Bars show the mean +/- 1 SEM of LV/field (*Ephb4$^{fl/fl}$*, n=6; *Ephb4$^{fl/fl}$ Ub^{ert2cre}*, n=10). (C) Skin sections were stained with anti-CD31 and anti-activated caspase 3 antibodies and Hoecsht to identify apoptotic EC. Examples of activated caspase 3-positive EC with fragmented nuclei in images of *Ephb4$^{fl/fl}$ Ub^{ert2cre}* sections are indicated with arrowheads. (D) Plot shows the percentage of apoptotic EC in individual CD31+ BV in skin of *Ephb4$^{fl/fl}$* and *Ephb4$^{fl/fl}$ Ub^{ert2cre}* embryos selected from multiple randomly chosen areas. Bars show the mean +/- 1 SEM of percentage apoptotic EC per vessel (*Ephb4$^{fl/fl}$*, n=11; *Ephb4$^{fl/fl}$ Ub^{ert2cre}*, n=17). ****, *P*<0.0001; Mann Whittney test.

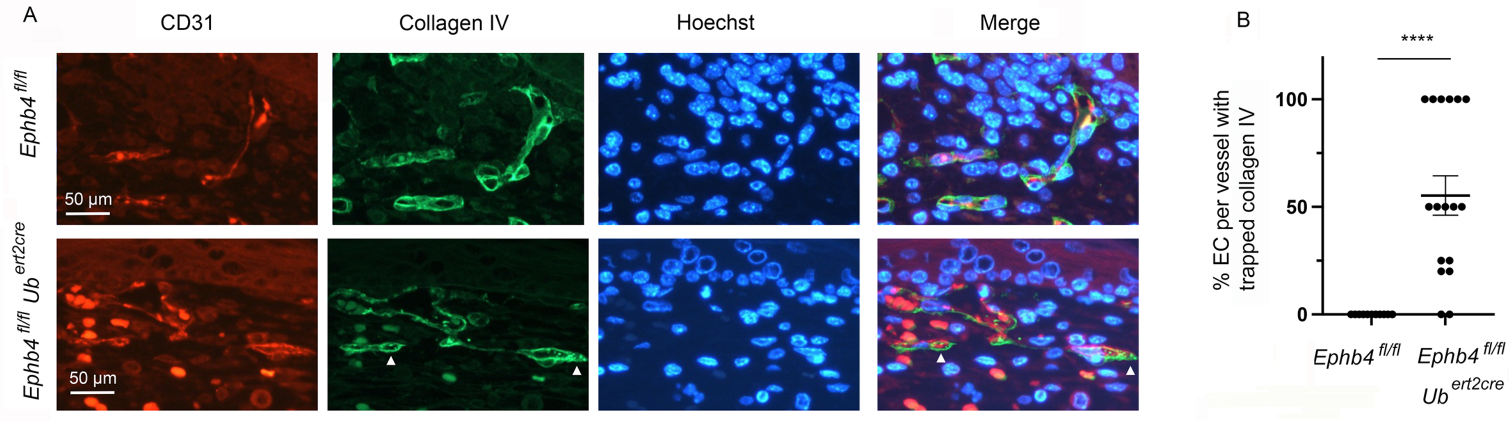

Supplemental Figure 3. Disruption of an exon 1 floxed *Ephb4* allele during developmental angiogenesis results in accumulation of collagen IV in EC. *Ephb4*$^{fl/fl}$ and *Ephb4*$^{fl/fl}$ *Ub*^{ert2cre} embryos in which exon 1 of *Ephb4* was floxed were administered TM at E13.5 and harvested at E18.5. (A) Skin sections were stained with anti-CD31 and anti-collagen IV antibodies and Hoechst. Note intracellular accumulation of collagen IV in EC of *Ephb4*$^{fl/fl}$ *Ub*^{ert2cre} embryos (examples highlighted with arrowheads). (B) Plot shows the percentage of EC with intracellular collagen IV punctae in individual CD31+ BV in skin of embryos selected from multiple randomly chosen areas. Bars show the mean +/- 1 SEM of percentage EC with collagen IV accumulation (*Ephb4*$^{fl/fl}$, n=11; *Ephb4*$^{fl/fl}$ *Ub*^{ert2cre}, n=17). ****, P<0.0001; Mann Whittney test.

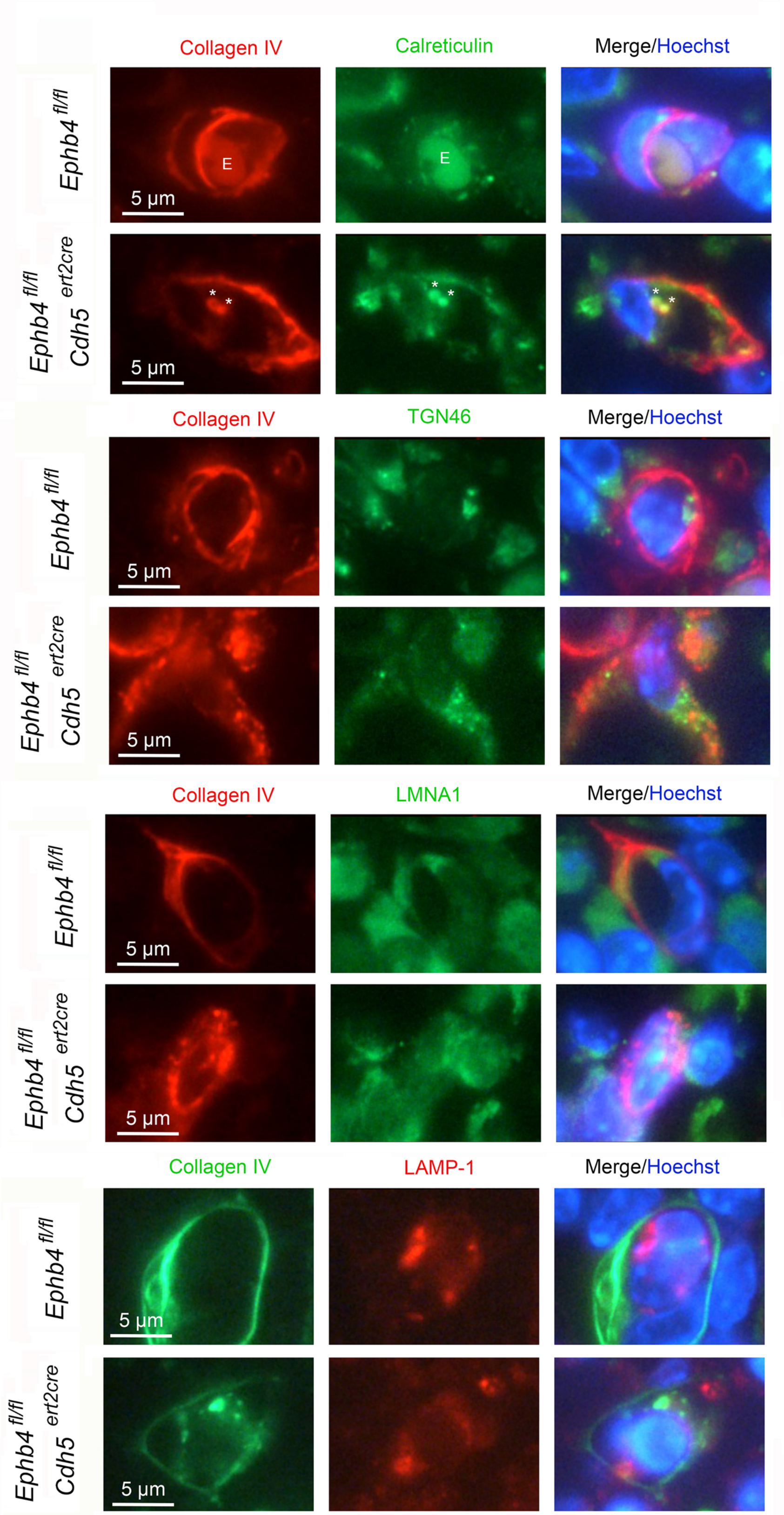

Supplemental Figure 4. Collagen IV is retained within the ER of induced EPHB4-deficient EC during developmental angiogenesis. TM was administered to *Ephb4*$^{fl/fl}$ and *Ephb4*$^{fl/fl}$ *Cdh5*^{ert2cre} embryos at E13.5 and embryos were harvested at E18.5. Skin sections were stained with anti-collagen IV antibodies and antibodies against calreticulin (ER), LMNA1 (ERGIC), TGN46 (Golgi) or LAMP-1 (lysosome). Representative images of individual BV are shown. Note colocalization of collagen IV punctae with calreticulin in *Ephb4*$^{fl/fl}$ *Cdh5*^{ert2cre} embryos (asterisks). Note lack of colocalization of collagen IV punctae with other organelle markers in *Ephb4*$^{fl/fl}$ *Cdh5*^{ert2cre} embryos. E, erythrocyte.

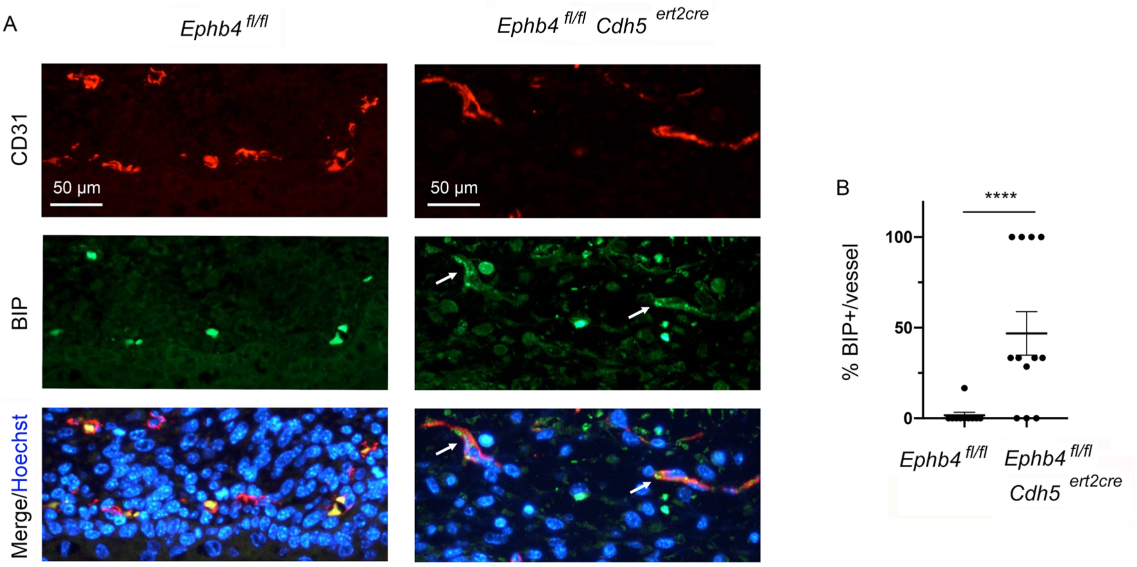

Supplemental Figure 5. Induction of BIP in induced EPHB4-deficient EC during developmental angiogenesis. TM was administered to *Ephb4*$^{fl/fl}$ and *Ephb4*$^{fl/fl}$ *Cdh5*^{ert2cre} embryos at E13.5 and embryos were harvested at E18.5. Skin sections were stained with anti-CD31 and anti-BIP antibodies. Note positive BIP staining in EC of *Ephb4*$^{fl/fl}$ *Ub*^{ert2cre} embryos (examples highlighted with arrows). (B) Plot shows the percentage of EC with BIP staining in individual CD31+ BV in skin of embryos selected from multiple randomly chosen areas. Bars show the mean +/- 1 SEM of percentage EC with BIP staining (*Ephb4*$^{fl/fl}$, n=10; *Ephb4*$^{fl/fl}$ *Cdh5*^{ert2cre}, n=12). ****, P<0.0001; Mann Whittney test.

Figure 6C

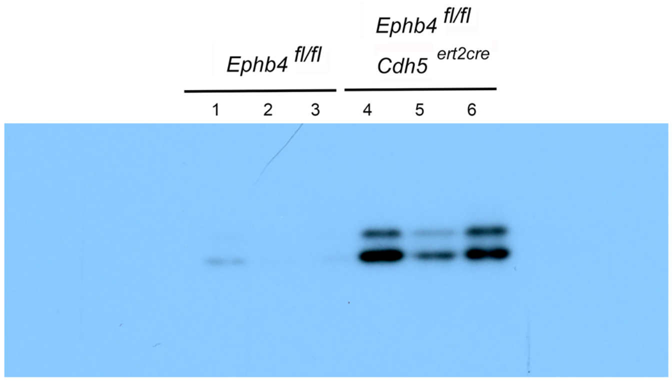

lysate blot pERK

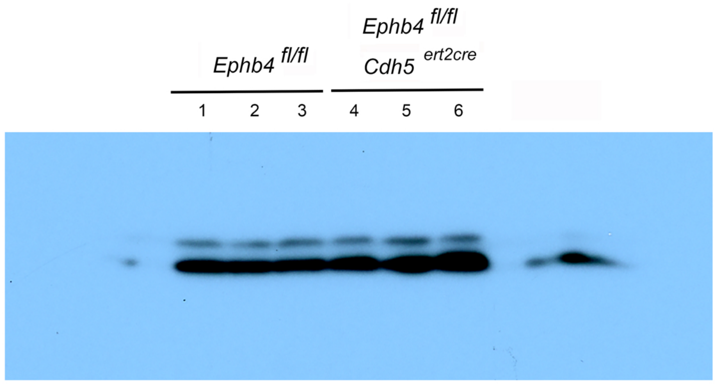

lysate blot ERK

Fig. 10 A

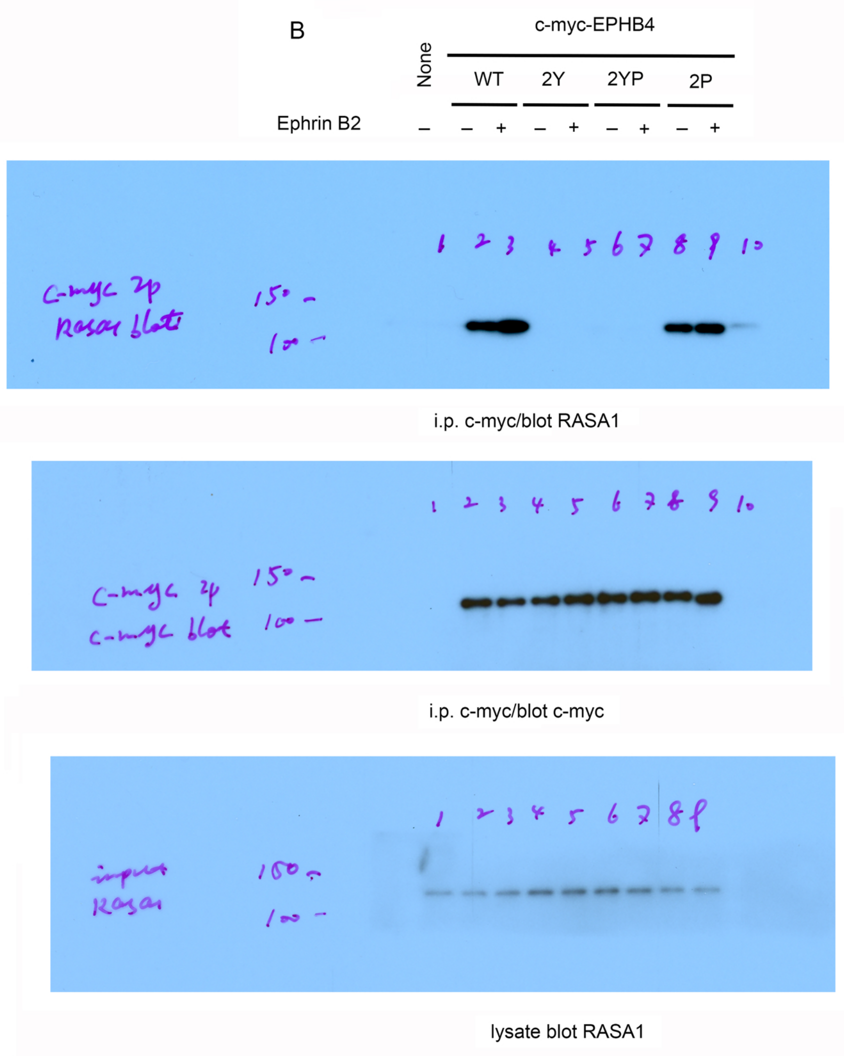

Fig. 10 B

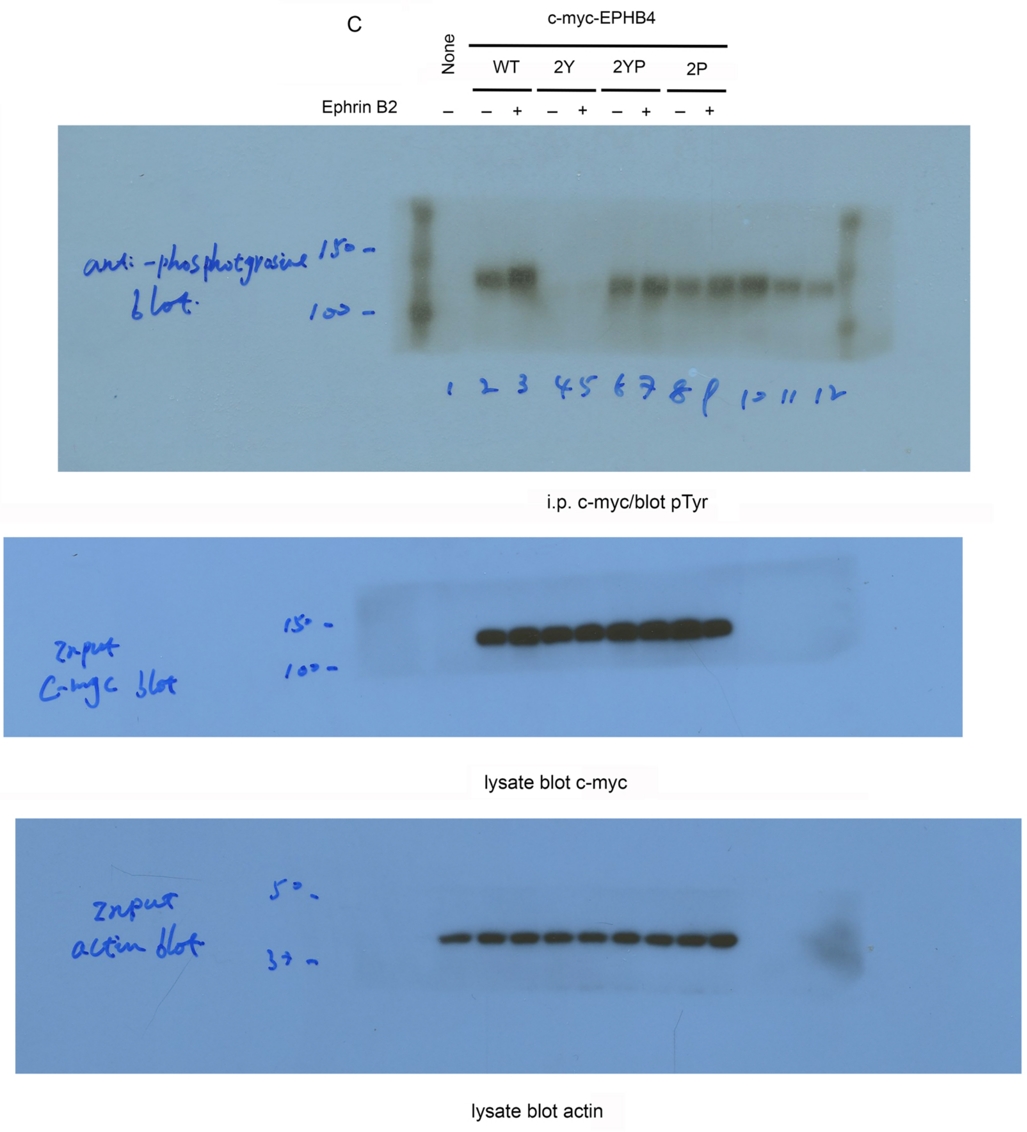

Supplement: Supplemental data [file jciinsight-7-156928-s114.pdf]
